# Metagenomic insights into S(0) precipitation in a terrestrial subsurface lithoautotrophic ecosystem

**DOI:** 10.3389/fmicb.2014.00756

**Published:** 2015-01-08

**Authors:** Trinity L. Hamilton, Daniel S. Jones, Irene Schaperdoth, Jennifer L. Macalady

**Affiliations:** ^1^Department of Geosciences, Penn State Astrobiology Research Center, The Pennsylvania State UniversityUniversity Park, PA, USA; ^2^Department of Earth Sciences, University of MinnesotaMinneapolis, MN, USA

**Keywords:** S(0), sulfide, Epsilonproteobacteria, Frasassi, Sqr, Sox, lithotrophy, autotrophy

## Abstract

The Frasassi and Acquasanta Terme cave systems in Italy host isolated lithoautotrophic ecosystems characterized by sulfur-oxidizing biofilms with up to 50% S(0) by mass. The net contributions of microbial taxa in the biofilms to production and consumption of S(0) are poorly understood and have implications for understanding the formation of geological sulfur deposits as well as the ecological niches of sulfur-oxidizing autotrophs. Filamentous Epsilonproteobacteria are among the principal biofilm architects in Frasassi and Acquasanta Terme streams, colonizing high-sulfide, low-oxygen niches relative to other major biofilm-forming populations. Metagenomic sequencing of eight biofilm samples indicated the presence of diverse and abundant Epsilonproteobacteria. Populations of *Sulfurovum*-like organisms were the most abundant Epsilonproteobacteria regardless of differences in biofilm morphology, temperature, or water chemistry. After assembling and binning the metagenomic data, we retrieved four nearly-complete genomes of *Sulfurovum*-like organisms as well as a *Sulfuricurvum* spp. Analyses of the binned and assembled metagenomic data indicate that the Epsilonproteobacteria are autotrophic and therefore provide organic carbon to the isolated subsurface ecosystem. Multiple homologs of sulfide-quinone oxidoreductase (Sqr), together with incomplete or absent Sox pathways, suggest that cave *Sulfurovum*-like Epsilonproteobacteria oxidize sulfide incompletely to S(0) using either O_2_ or nitrate as a terminal electron acceptor, consistent with previous evidence that they are most successful in niches with high dissolved sulfide to oxygen ratios. In contrast, we recovered homologs of the complete complement of Sox proteins affiliated Gammaproteobacteria and with less abundant *Sulfuricurvum* spp. and *Arcobacter* spp., suggesting that these populations are capable of the complete oxidation of sulfide to sulfate. These and other genomic data presented here offer new clues into the physiology and genetic potential of the largely uncultivated and ecologically successful cave *Sulfurovum*-like populations, and suggest that they play an integral role in subsurface S(0) formation.

## Introduction

The subsurface of Earth remains one of the least explored habitats. Dark terrestrial ecosystems such as caves are rarely studied in part due to low biomass and physical inaccessibility. Sulfidic caves form in limestone rocks where anoxic, sulfide-rich groundwater interacts with oxygenated surface recharge and cave air at the water table. Deep subsurface, sulfidic cave systems such as Frasassi and Acquasanta Terme in Italy reach up to 500 m below ground surface and have been the target of multiple investigations in the past decade due to their unique biogeochemistry and relative accessibility to human exploration (Galdenzi and Maruoka, 2003; Macalady et al., 2006, 2007, 2008; Galdenzi et al., 2008; Jones et al., 2010). The Frasassi subsurface ecosystem is sustained exclusively by microbial chemosynthesis taking place near the surface of the perennially sulfidic and microoxic (2–25 μM dissolved O_2_) aquifer. Filamentous Gamma- and Epsilonproteobacteria are dominant populations in conspicuous, sulfur-rich, white biofilms in the cave waters (Macalady et al., 2008). Collectively, these geochemical and microbiological features make Frasassi a promising model system for investigating microbial interactions with zero valent sulfur [S(0)], an important solid-phase intermediate in the sulfur cycle.

S(0) can be formed by both chemical and biological sulfur oxidation or reduction. It is found as an intermediate redox species in anoxic water columns (e.g., Luther et al., 1991), marine sediments (e.g., Troelsen and Jørgensen, 1982), marshes (e.g., Zopfi et al., 2004), terrestrial geothermal springs (e.g., Nordstrom et al., 2005), deep-sea hydrothermal vents (e.g., Taylor and Wirsen, 1997; Embley et al., 2007), supraglacial sulfur springs (Grasby et al., 2003), and sulfidic caves (Boston et al., 2006; Galdenzi et al., 2008). Conspicuous accumulation of S(0) at sites such as pelagic oxygen minimum zones, hydrothermal vents and sediment surfaces has been attributed to microbially-mediated sulfide oxidation (Taylor et al., 1999; Wirsen et al., 2002; Jansen et al., 2009; Lavik et al., 2009). In natural waters, chemical sulfide oxidation occurs on the order of hours to days—the half-life of sulfide ranges from 16 to 50 h in fresh water (Zhang and Millero, 1993 and references therein). The relatively slow abiotic reaction kinetics allow biological S oxidation to compete with abiotic rates. Chemical oxidation of dissolved sulfide is slower in the absence of metals, and decreases with decreasing concentrations of sulfide and oxygen (Millero et al., 1987). Biological rates are typically higher than abiotic rates when cell concentrations exceed ~10^4^ cells cm^−3^, due to the presence of abundant enzymes that catalyze steps in sulfur oxidation (Jannasch et al., 1991; Canfield, 2004). Although much remains to be learned about abiotic sulfur oxidation kinetics and reactions, microbial activity appears to control sulfur speciation under conditions relevant to a wide range of natural environments, including the microoxic, low-metal, highly-colonized subsurface environments of the Frasassi cave systems and the nearby thermal Grotta Nuova di Rio Garrafo near Acquasanta Terme.

Elemental sulfur [S(0)] is a key intermediate in microbial sulfide oxidation and many microbes are known to precipitate S(0) either inside or outside of the cell. Species that produce internal S(0) include both phototrophs and lithotrophs, many of which can use S(0) as an electron donor or acceptor when supplies from the environment are limiting or absent. For instance, *Beggiatoa* spp. can reduce intracellular S(0) when more favorable electron acceptors are unavailable (Nelson and Castenholz, 1981; Schmidt et al., 1987). Phototrophic S-oxidizing members of the Chlorobi, Ectothiorhodospira, and Cyanobacteria produce extracellular S(0), as do the chemolithotrophic S-oxidizers *Arcobacter sulfidicus* and certain species of *Thiobacillus*, *Acidothiobacillus*, and *Thioalkalivibrio* (Kleinjan et al., 2003). By analogy with sulfur oxidizers that store S(0) internally, extracellular S(0) could function as energy or electron storage. Unlike internal S(0), extracellular S(0) is in theory accessible to other organisms, though it has been shown that Chlorobi (e.g., *Chlorobaculum tepidum*) access their own extracellular S(0) as an electron donor for photosynthesis and growth (Chan et al., 2008a). The physicochemical and genetic/biochemical mechanisms by which Chlorobi access external S(0) are still poorly understood. Alternative to the idea that external S(0) is an important energy/electron reserve for the populations that produce it, microbes may also excrete S(0) because it is an unneeded waste product produced in energy metabolism and/or detoxification reactions (Sievert et al., 2007). Extracellular S(0) cycling is poorly studied relative to intracellular storage, in part because it is challenging to link S(0) production and consumption with specific microorganisms in mixed populations or environmental samples. However, extracellular S(0) precipitation—mediated by biological mechanisms—may be of greater geologic importance because of the potential for high production rates. Therefore, we have strong motivation to investigate the ecology, physiology, and microbe-mineral interactions of microorganisms that may be major producers of extracellular S(0) in the environment.

In the presence of electron acceptors such as oxygen or nitrate, sulfide can be oxidized completely to sulfate. However, oxidation may also occur in gradient or mixing zones with low oxygen (and nitrate) availability, resulting in sulfide incompletely oxidized to S(0). The resulting S(0) can have several fates—it can be further oxidized to sulfate if sufficient electron acceptors become available, it can serve as an electron acceptor, or it can be disproportionated. Environmental concentrations of S(0) reflect the net balance of production and consumption. S(0) turnover times have been measured in days (e.g., up to 66 days in Black sea sediments, by Zopfi et al., 2004). In order for S(0) to be deposited on geologic time scales, turnover times must be much longer (e.g., 10^6^ years), requiring either high production and negligible consumption or a physical mechanism for sequestering S(0) from further biological transformations. Although the most conspicuous modern environments of S(0) deposition are associated with volcanism, it is estimated that only <5–10% of geologic S(0) deposits are volcanogenic (Ivanov and Starkey, 1968; Trudinger, 1982). Most economic S(0) deposits are epigenetic, formed in the subsurface and associated with gypsum/anhydrite sand carbonate sedimentary sequences. These economic S(0) deposits are considered to be biogenic based on S isotopic evidence (Ruckmick et al., 1979; Trudinger, 1982). Their relatively light isotopic composition reflects microbial reduction of gypsum-derived sulfate to sulfide, coupled with oxidation of sedimentary organic carbon including hydrocarbons (Hill, 1995). The oxidation of sulfide to S(0) under microoxic conditions (i.e., below the water table) could be biotic, but this has not been conclusively demonstrated.

Enigmatic Epsilonproteobacteria from the globally distributed and largely uncultivated provisional “Thiovulgaceae” clade (Campbell et al., 2006), are major populations in Frasassi biofilms (Macalady et al., 2006, 2008; Jones et al., 2010), and are widely distributed in other environments (Rossmassler et al., 2012). In the Frasassi cave system, *Sulfurovum*-like organisms are abundant in niches with high sulfide and low oxygen supply ratios (Macalady et al., 2008), redox conditions which should lead to S(0) accumulation due to limited availability of electron acceptors. Unlike many lithotrophic S-oxidizers (e.g., *Beggiatoa* spp., *Thiothrix* spp.), pure cultures of Epsilonproteobacteria do not appear to store intracellular S(0) (Engel et al., 2004), a characteristic that may have important implications for the fate of S(0) in the environment. Studies of existing Epsilonproteobacteria isolates are suggestive but do not offer conclusive insights about S(0) production in ecologically successful, uncultivated groups. All of the 8 isolates within the largely uncultivated “Thiovulgaceae” clade are mesophilic, microaerobic autotrophs that use oxygen or nitrate/nitrite as electron acceptors (Wirsen and Jannasch, 1978; Gevertz et al., 2000; Inagaki et al., 2003, 2004; Kodama and Watanabe, 2003, 2004; Nakagawa et al., 2005a). With the exception of *Nitratifractor salsuginis*, all of the isolates are capable of oxidizing forms of reduced sulfur— sulfide, thiosulfate or S(0)—for growth (Campbell et al., 2006). Outside the “Thiovulgaceae,” extracellular S(0) formation has been studied in enrichment cultures of *Candidatus* Arcobacter sulfidicus, which form remarkable S(0) filaments and, given their apparent productivity—single cells form S(0) filaments at a linear rate of 3 μm min^−1^, resulting in material that is 82% S by weight—are possibly the fastest known S(0)-producers (Taylor and Wirsen, 1997). The ecological function of filamentous S(0) formation is not known. Current hypotheses suggest that it is formed as the result of rapid sulfide detoxification and/or as a holdfast to maintain position in sulfide gradients where both sulfide and oxidants are optimally balanced (Sievert et al., 2007).

While the predominant mechanism of S(0) precipitation in sulfidic caves such as Frasassi and Grotta Nuova del Rio Garrafo (Acquasanta Terme) is not known, biotic processes are likely important given the conspicuous microbial life near the surface of the cave aquifers (i.e., Engel et al., 2004; Macalady et al., 2006, 2008; Jones et al., 2010). The cave populations have <91.5% 16S rRNA sequence similarity to the two cultivated representatives (both vent-dwelling *Sulfurovum* spp.), and the vast majority of strains in this clade have not yet been cultivated. Here, we consider the energetics of sulfide oxidation under the conditions observed in the cave waters, micro-oxic and low in nitrate, and employ metagenomic sequencing to examine the role of Epsilonproteobacteria in S(0) formation. We recovered several near complete genomes of Epsilonproteobacteria affiliated with *Sulfurovum*-like organisms and one from a *Sulfuricurvum* spp. The genomes (and metagenomes) provide new insights into the physiology of these uncultivated organisms and their potential role in the global sulfur, carbon and nitrogen cycles.

## Materials and methods

### Site description, sample collection and geochemistry

Biofilms for this study were collected from previously described sample locations (Macalady et al., 2006, 2008; Jones et al., 2010) in the sulfide-rich Frasassi (43.3983 N, 12.9621 E) and Grotta Nuova del Rio Garrafo (42.753 N, 13.411 E) caves in the Marche Region, Italy. The Frasassi and Rio Garrafo caves in this study are actively forming in Jurassic limestones (Calcare Massiccio and Maiolica Fms.) and a marly Eocene limestone (Scalia Rossa Fm.), respectively. In both caves, sulfidic ground waters from deep-seated anoxic aquifers emerge as streams and lakes at the cave water table, and can be reached by technical caving routes. Cave waters at Frasassi are slightly saline (conductivity 1000–3500 μS/cm), with sulfide concentrations up to 600 μM and constant temperature (13–14°C) (Macalady et al., 2006; Galdenzi et al., 2008). The sulfidic stream in Grotta Nuova del Rio Garrafo is warmer and saltier, with specific conductivities over 10,000 μS/cm, temperatures up to 45°C, and sulfide concentrations as high as 800 μM (Galdenzi et al., 2010; Jones et al., 2010). Sulfidic waters in both cave systems are perennially low in dissolved oxygen (<30 uM), with nitrate concentrations consistently below detection limits (~7 nM) and ammonium concentrations ranging from 35 to 74 μM (Macalady et al., 2006; Jones et al., 2010).

Biofilms included in the present study were collected between June 2006 and June 2010 from microaerophilic sulfidic cave streams at Grotta Sulfurea (GS), Pozzo dei Cristalli (PC), and Fissure Spring (FS) at Frasassi and from a sulfidic stream in Grotta Nuova di Rio Garrafo in Acquasanta Terme (AS) (Table 1; Figure S1). Conductivity, pH and temperature of the streams were measured in the field at the time of sample collection using sensors attached to a 50i multimeter (WTW, Weilheim, Germany). Dissolved sulfide and oxygen concentrations were measured in the field using a portable spectrophotometer (Hach Co., Loveland, CO, USA) using the methylene blue and indigo carmine methods, respectively (Hach methods 690 and 8316). Duplicate sulfide analyses were within 1% of each other. Replicate oxygen analyses were within 20% of each other. Nitrate, nitrite, ammonium and sulfate were measured at the Osservatorio Geologico di Coldigioco Geomicrobiology Lab using a portable spectrophotometer within 12 h of collection according to the manufacturer's instructions (Hach Co.) All other water samples were filtered (0.2 μm) into acid-washed polypropylene bottles and stored at 4°C or −20°C until analyzed.

**Table 1:** **Geochemical data^a^ for the cave waters where biofilms were sampled in the present study**.

**Location^b^**	**Collection date**	**Cond. (uS/cm)**	**pH**	**Temp. (°C)**	**O^c,d^_2_ (μM)**	**∑H_2_S (μM)**
Acquasanta Terme (AS07-7)	1 Jun 07	10640	6.38	44.1	3.1	851.6
Fissure Spring (FS06-10)	25 Jun 06	2740	7.25	14.3	1.5	436.7
Fissure Spring (FS08-3)	5 Jun 08	1800–2600	7.03–7.11	14.6	3.9	346.1
Grotto Sulfurea (GS09-5)	19 May 09	1721	7.30	13.3	22.5	78.6
Grotto Sulfurea (GS10-10)	4 Jun 10	1609	7.37	13.4	29.7	18.4
Pozzo dei Cristalli (PC08-3)	26 May 08	2830	7.31	13.4	BD	588.3
Pozzo dei Cristalli (PC08-64)	24 Jun 08	2930	7.16	13.4	14.5	421.1
Pozzo dei Cristalli (PC08-66)	24 Jun 08	2930	7.16	13.4	14.5	421.1

a Nitrate was below the method detection limit at all sites (method detection limit, NO_3−_ = 0.7 μM).

b Sample IDs in parentheses.

c BD, below detection limit.

d Method detection limit, O_2_ = 1 μM.

### Elemental analyses

Samples collected for elemental analyses were stored at 4°C immediately upon collection and dried at 70°C within 24 h. Samples for S and C analyses were prepared as described previously (Jones et al., 2008). Elemental analysis for carbon and sulfur was performed at the Agricultural Analytical Services Laboratory at Pennsylvania State University, where total carbon was determined by combustion in a Fisons NA 1500 Elemental Analyzer and sulfur was determined by microwave digestion (Miller, 1998).

### DNA extraction

Environmental DNA was extracted as previously described (Macalady et al., 2008), using phenol-chloroform extraction after first diluting the RNAlater (Ambion/Applied Biosystems, Foster City, CA, USA) preserved sample with three parts phosphate-buffered saline to one part sample. Sample quality and concentration were checked on a 1% agarose gel using the HiLo DNA Marker (Bionexus, Oakland, CA).

### Metagenome sequencing and assembly

DNA fragments were prepared by the Joint Genome Institute following their standard library generation protocol for Illumina 2000 platform and sequenced on a HiSeq 2000 system. Reads were trimmed and assembled according to standard JGI protocols. Briefly, raw Illumina metagenomic reads were screened against Illumina artifacts with a sliding window with a kmer size of 28 and a step size of 1. Screened reads were trimmed from both ends using a minimum quality cutoff of 30—reads with 3 or more N's or with average quality score of less than Q20 were removed. In addition, reads with a minimum sequence length of <50 bps were removed. Trimmed, screened, paired-end Illumina reads were assembled using SOAPdenovo v1.05 (http://soap.genomics.org.cn/soapdenovo.html) at a range of kmers (81,85,89,93,97,101). Default settings for all SOAPdenovo assemblies were used (options “-K 81 -p 32 -R -d 1”). Contigs generated by each of the eight assemblies were de-replicated using in-house (JGI) Perl scripts. Contigs were then sorted into two pools based on length. Contigs smaller than 1800 bp were assembled using Newbler (Life Technologies, Carlsbad, CA) in attempt to generate larger contigs (flags: -tr, -rip, -mi 98, -ml 80). All assembled contigs larger than 1800 bp, as well as, the contigs generated from the final Newbler run were combined using minimus 2 (flags: -D MINID = 98 -D OVERLAP = 80) (AMOS:, http://sourceforge.net/projects/amos). Read depths were estimated based on read mapping with BWA using default parameters (http://bio-bwa.sourceforge.net/; Li and Durbin, 2010).

### Metagenome annotation and binning

Annotation of contigs was performed using the Joint Genome Institute's Integrated Microbial Genomes with Microbiomes-Expert Review (IMG/M-ER) pipeline (Markowitz et al., 2010). Details of gene annotation are available from the JGI website (http://img.jgi.doe.gov/w/doc/about_index.html). Phylum- and class-level assignment of all annotated sequences (prior to binning) was assigned using the best BLASTX hit with *e*-value scores less than 1e^−4^ and quality coverage of at least 75%. Assembled contigs ≥2500 kb were binned using an emergent self-organizing map based on tetranucleotide frequency of the contigs, resulting in taxonomically-resolved clusters of scaffolds containing individual genomes (Dick et al., 2009). Tetranucleotide frequency was calculated as previously described (Hamilton et al., 2014) with a custom perl script (available at https://github.com/bovee/Ochre) and visualized with the Databionic-ESOM Tools (http://databionic-esom.sourceforge.net) using parameters described in Dick et al. (2009). Well-defined Epsilonproteobacteria bins [or “genomic bins” (Voorhies et al., 2012)] were identified using taxonomic affiliation of predicted genes based on the best BLAST match. For inclusion of a scaffold in the genomic bin, greater than 50% of the genes on the scaffold had to have best matches, via BLAST, to Epsilonproteobacteria. Genomic bins were further curated with coverage and GC content. To improve assembly, paired reads mapping to each genomic bin were reassembled as described previously (Hug et al., 2013) using Velvet (Zerbino and Birney, 2008) or IDBA_UD under default parameters (Peng et al., 2012). The assembly with the longest contigs, highest N50, and lowest number of contigs was selected as the final assembly for each bin. Genome completeness was evaluated using phylogenetic marker genes (Table S1) identified by annotation and using genes identified with Phyla-AMPHORA (Wang and Wu, 2013). All scaffolds encoding *sox* genes were manually inspected for %GC, tetranucleotide frequency, genomic context and best BLASTP/X hits of all genes to verify the *sox*-containing contigs were correctly binned.

### 16S rRNA gene reconstruction

Near full-length 16S rRNA sequences were re-constructed from Illumina sequencing reads from each metagenome using EMIRGE (Miller et al., 2011). EMIRGE was run for 100 iterations with default parameters designed to merge reconstructed 16S rRNA genes if candidate consensus sequences share ≥97% sequence identity in any iteration. The non-redundant SILVA SSU reference database version 111 (http://www.arb-silva.de/) was the starting database. Sequences with an estimated abundance of less than 0.01% were removed from further analyses. Potential chimeras were identified with UCHIME (Edgar et al., 2011) using Mothur (ver 1.32.1; Schloss et al., 2009) and removed from further analyses. Taxonomic assignment of the EMIRGE-reconstructed 16S rRNA sequences was performed using BLAST and ARB (Ludwig et al., 2004).

### Phylogenetic analyses

The phylogenetic position of bacterial 16S rRNA genes was evaluated by approximate likelihood-ratio tests (Anisimova and Gascuel, 2006) as implemented in PhyML v. 3.0 (Guindon and Gascuel, 2003). Sequences were aligned with Mega v 6.0 (Tamura et al., 2013) and the best evolutionary model was determined using jModeltest (version 2.1.1, Darriba et al., 2012). Maximum likelihood reconstructions used the General Time Reversible substitution model and gamma-distributed rate variation with a proportion of invariable sites as recommended by jModeltest. Phylograms were rate-smoothed using the multidimensional version of Rambaut's parameterization as implemented in PAUP v. 4.0 (Swofford, 2001) as previously described (Meuser et al., 2013).

16S rRNA sequences often fail to assemble into larger contigs and scaffolds in metagenomic studies. In addition, 16S rRNA sequences resulting from independent re-construction of these genes from metagenomic sequencing data using programs like Phyloshop (Shah et al., 2011) or EMIRGE (Miller et al., 2011) are difficult to assign to bins separated by genomic signatures such as tetranucleotide frequency or %GC-content. To overcome these obstacles, a subset of single-copy ribosomal proteins (*n* = 19) (Table S2) were used for taxonomic assignment of each *Sulfurovum*-like genomic bin as previously described (Hug et al., 2013; Hamilton et al., 2014) and to verify the taxonomic assignment of the *Sulfuricurvum* genomic bin. Sequences from each genomic bin were added to the reference database supplied with the Phyla-AMPHORA package. Reference datasets were further populated with sequences of closely related organisms mined from genome sequences in the NCBI databases and JGI IMG-M using BLASTP and verified by genomic context. Each protein was aligned individually using ClustalX (version 2.1) using the Gonnet 250 protein substitution matrix and default gap extension and opening penalties (Larkin et al., 2007). Alignments were manually curated and the best evolutionary model was determined using ProtTest (version 3, Darriba et al., 2011). Individual alignments were then concatenated and a maximum likelihood phylogeny was calculated using PhyML (Guindon and Gascuel, 2003) with one thousand bootstrap replicates.

Full-length sequences of select functional genes (e.g., *sqr*, *napA*) were used for phylogenetic analyses. For each functional gene, datasets included sequences identified by IMG annotation and BLAST analysis (BLASTX/P) with the following criteria: *e*-value scores less than 1e^−4^ and quality coverage of at least 75%. Amino acid sequences for each functional gene were aligned with ClustalX (version 2.1) as described above and manually curated. For DsrA and DsrB, sequences were aligned and alignments were concatenated prior to phylogenetic analyses. The phylogeny of each functional gene or gene set (DsrAB) was evaluated using PhyML (Guindon and Gascuel, 2003) with 1000 bootstrap replicates using the best evolutionary model as identified by ProtTest (version 3, Darriba et al., 2011). For identification and quantification of functional genes, full-length sequences were mined from the metagenomes using functional annotation and BLAST analysis using the same criteria described above. Multiple query sequences for BLAST searches were chosen to sample the diversity of organisms present in the sample. Phylum- and genus-level affiliations were assigned based on the best BLASTP hit to each functional gene for all alignments covering greater than 70% of the total query, with *e*-value scores greater than 1e^−4^ and a bit score >100. Abundance for each functional gene was normalized to gene length and the total number of reads per dataset.

### Nucleotide sequence accession numbers

Access to the metagenomes is provided by the DOE Joint Genome Institute (JGI) at the Integrated Microbial Genome (IMG-M) site: https://img.jgi.doe.gov/cgi-bin/m/main.cgi (Table S3). Raw sequence reads of all samples were deposited at the NCBI Short Read Archive (SRA) and can be accessed under the accession numbers SRR1559028, SRR1559230, SRR1559353, SRR1560064, SRR1560266, SRR1560848, SRR1560849, and SRR1560850. Genomic bin sequences—AS07-7 *Sulfurovum*-like, PC08-66 *Sulfuricurvum*, PC08-66, *Sulfurovum*-like, FS06-10 *Sulfurovum*-like, and FS08-3 *Sulfurovum*-like—were deposited at DDBJ/EMBL/GenBank under the accession numbers JQIP00000000, JQIQ00000000, JQIR00000000, JQIS00000000, and JQIT00000000, respectively; and the versions described in the this paper are JQIP01000000, JQIQ01000000, JQIR01000000, JQIS01000000, and JQIT01000000, respectively. EMIRGE-reconstructed 16S rRNA gene sequences have been deposited in GenBank under the accession numbers KM410305—KM410928.

## Results and discussion

### S(0) formation

Oxidation of sulfide during aerobic respiration or under denitrifying conditions can lead to the formation of either sulfate or S(0) Equations (1–8). Complete oxidation to sulfate Equations (1, 3, 5, 7) is energetically favored regardless of electron acceptor, while incomplete oxidation to S(0) requires less electron acceptor (O_2_ or nitrate) Equations (2, 4, 6, 8). Both oxygen-limiting and nitrate-limiting concentrations have been implicated as favoring S(0) formation during sulfide oxidation *in situ*. For instance, in an autotrophic denitrifying community from anaerobic sludge, nitrate-limitation favored the production of S(0) while complete oxidation of sulfide to sulfate was observed when ample nitrate was supplied (Cardoso et al., 2006). The relative importance of genetic or ecological mechanisms controlling this phenomenon are not well understood and, importantly, have not been characterized in pure culture. At the oxic-anoxic interface, the cave waters are both oxygen- and nitrate-limited (with respect to sulfide) (Table 1), and the lack of sufficient electron acceptors presumably favors the production of S(0). Accordingly, pH microsensor measurements found that no measurable acid production occurs within Frasassi stream biofilms, which indicates that S(0) and not sulfate is the primary end product of sulfide oxidation in the streams (Jones et al., unpublished data). Despite observations of perennially low levels of nitrate in all sample sites, these experiments do not indicate if nitrate or oxygen is the preferred electron acceptor. Furthermore, the rates of nitrate production and consumption *in situ* have not been characterized.

(1) $$\begin{array}{l} {\text{H}}_{2}\text{S}+2{\text{O}}_{2}\to {\text{SO}}_{4}^{2-}+2{\text{H}}^{+}\\ \;\Delta G^{{{}^{\circ}}^{\prime }}=-790.0\text{kJ}/\text{reaction} \end{array}$$

(2) $$\begin{array}{l} {\text{H}}_{2}\text{S}+0.5{\text{O}}_{2}\to {\text{S}}^{0}+{\text{H}}_{2}\\ \;\Delta {\text{G}}^{{{}^{\circ}}^{\prime }}=-420.7\text{kJ}/\text{reaction } \end{array}$$

(3) $$\begin{array}{l} {\text{H}}_{2}\text{S}+1.6{\text{NO}}_{3}^{-}\to {\text{SO}}_{4}^{2-}+0.8{\text{N}}_{2}+{\text{H}}_{2}\text{O}\\ \;\Delta {\text{G}}^{{{}^{\circ}}^{\prime }}=-746.3\text{kJ}/\text{reaction} \end{array}$$

(4) $$\begin{array}{l} {\text{H}}_{2}\text{S}+0.33{\text{NO}}_{3}^{-}\to {\text{S}}^{0}+0.16{\text{N}}_{2}+{\text{H}}_{2}\text{O}\\ \;\Delta {\text{G}}^{{{}^{\circ}}^{\prime }}=-196.8\text{kJ}/\text{reaction} \end{array}$$

(5) $$\begin{array}{l} {\text{H}}_{2}\text{S}+{\text{NO}}_{3}^{-}+{\text{H}}_{2}\text{O}\to {\text{SO}}_{4}^{2-}+{\text{NH}}_{4}^{+}\\ \;\Delta {\text{G}}^{{{}^{\circ}}^{\prime }}=-460.8\text{kJ}/\text{reaction} \end{array}$$

(6) $$\begin{array}{l} {\text{H}}_{2}\text{S}+{\text{NO}}_{3}^{-}+8{\text{H}}^{+}\to {\text{S}}^{0}+{\text{NH}}_{4}^{+}+3{\text{H}}_{2}\text{O}\\ \;\Delta {\text{G}}^{{{}^{\circ}}^{\prime }}=-125.8\text{kJ}/\text{reaction} \end{array}$$

(7) $$\begin{array}{l} {\text{H}}_{2}\text{S}+4{\text{NO}}_{3}^{-}\to {\text{SO}}_{4}^{2-}+4{\text{NO}}_{2}^{-}+2{\text{H}}^{+}\\ \;\Delta {\text{G}}^{\circ^{\prime }}=-499.4\text{kJ}/\text{reaction} \end{array}$$

(8) $$\begin{array}{l} {\text{H}}_{2}\text{S}+{\text{NO}}_{3}^{-}\to {\text{S}}^{0}+{\text{NO}}_{2}^{-}+{\text{H}}_{2}\text{O}\\ \;\Delta {\text{G}}^{\circ^{\prime }}=-135.1\text{kJ}/\text{reaction} \end{array}$$

### Metagenomes of sulfur-rich cave biofilms

Sulfur-rich biofilms are common in Frasassi and Rio Garrafo cave waters, and previous FISH population counts indicated that filamentous *Sulfurovum*-like Epsilonproteobacteria are the dominant populations in biofilms colonizing water with high dissolved sulfide to oxygen ratios (Macalady et al., 2008). Biofilms with abundant Epsilonproteobacteria contain ~ 45% total sulfur by mass, substantially more than sediments immediately underlying the biofilms at the same sample locations (Figure S2). This pattern suggests that sulfur oxidation in biofilms colonizing the sediment-water interface or water column is a more important source of sulfur than sulfate reduction in the underlying, anoxic sediments. Therefore, we sequenced eight metagenomes derived from sulfur-rich cave biofilms dominated by a variety of gamma- and epsilonproteobacterial populations.

**Figure 1:**
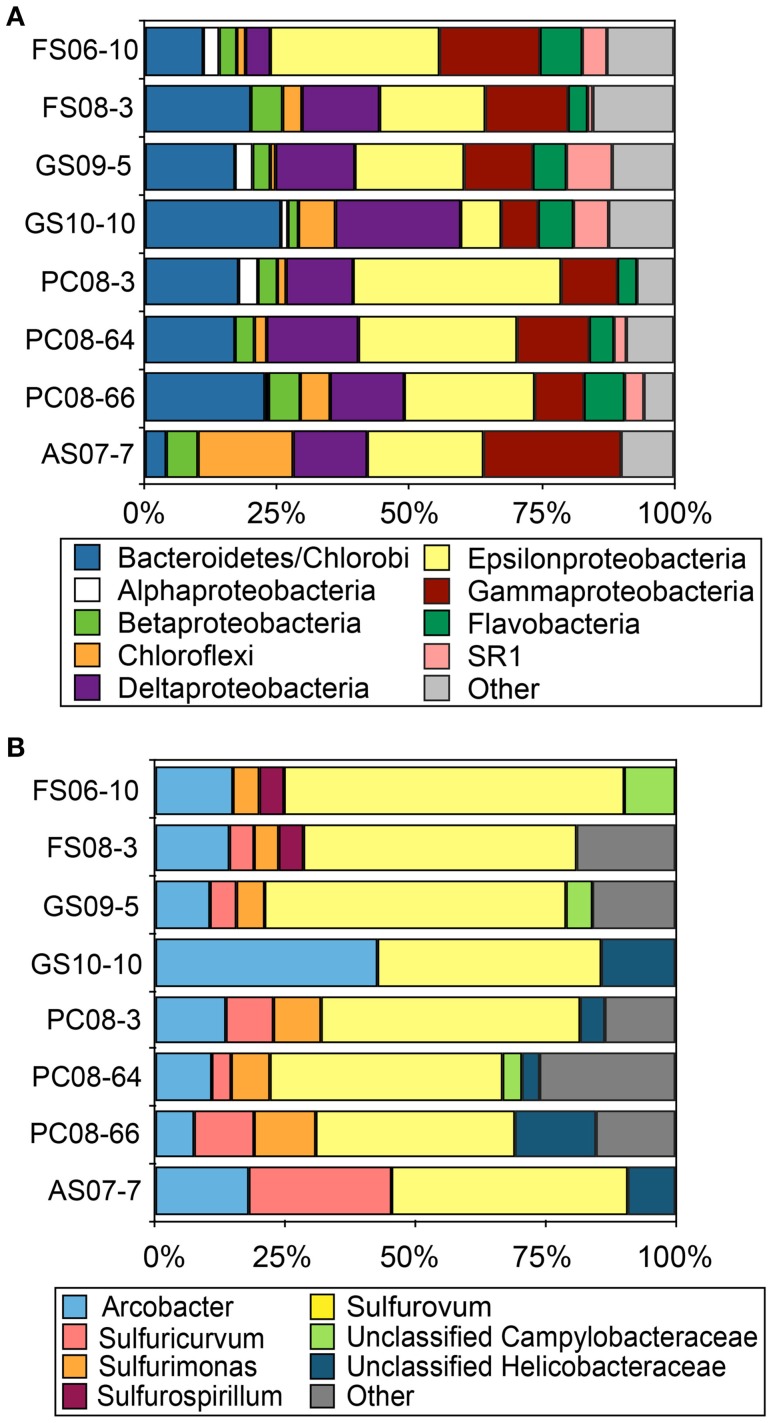
**Taxonomic affiliation of EMIRGE-reconstructed 16S rRNA sequences**. Taxonomic affiliation assigned at the phylum-level (except the Proteobacteria which are represented by class) **(A)** and at the genus level for Epsilonproteobacteria **(B)**. Other indicates all phyla or genus represented by less than 2.5% of the total.

Reconstruction of full-length 16S rRNA sequences from the Illumina metagenomic reads with EMIRGE resulted in 624 OTUs. The majority of these sequences were affiliated with Bacteriodetes, Delta-, Gamma- and Epsilonproteobacteria in all metagenomes with the exception of AS where Chloroflexi-affiliated 16S rRNA sequences accounted for ~18% of the total. (Figure 1A). Assignment of the EMIRGE-reconstructed epsilonproteobacterial 16S rRNA at the genus-level (based on BlastN analyses and phylogenetic placement) indicated sequences most closely related to *Sulfurovum* spp., i.e., *Sulfurovum* sp. NBC37-1 and *Sulfurovum* sp. AR, were most abundant in all metagenomes except AS (Figure 1B). Sequences assigned to the genus *Arcobacter* were also abundant is most of the metagenomes. The AS sample contained the largest number of *Sulfuricurvum*-like sequences (~25% of the total). The abundance of *Sulfurovum*-like and *Arcobacter*-like sequences is consistent with previous analyses of biofilm communities in the Frasassi and Acquasanta Terme cave streams (Macalady et al., 2006, 2008; Jones et al., 2010).

The eight metagenomes were assembled *de novo* resulting in datasets which ranged in size from 261,777 contigs and ~181 Mbp to 770,535 contigs and ~581 Mbp, with maximum contig lengths from 118,693 to 356,075 bp and N50 values ranging between 1 and 2.5 kb (Table S3). Overall, the majority of assembled sequences in all samples were assigned to bacteria (Figure S3). Archaeal and eukaryota-affiliated contigs were rare except in the AS (12% archaea) and GS samples (12–13% eukarya). Community composition based on assembled metagenomic sequences revealed a similar distribution to the EMIRGE-reconstructed 16S rRNA sequences (Figure 1A; Figure S4). Of the assembled sequences assigned to Epsilonproteobacteria, those affiliated with *Sulfurovum* were abundant in all biofilm samples. *Sulfuricurvum*-affiliated sequences were abundant in FS08-3 and PC-8-64 (Figure S5). We also examined the phylogenetic position and abundance of epsilonproteobacterial-affiliated single-copy ribosomal SP3 protein sequences from the metagenomes compared to those from complete or nearly complete genomes (Figure S6). Consistent with the 16S rRNA results, SP3 protein sequences affiliated with characterized *Sulfurovum* spp. (i.e., *Sulfurovum* sp. NBC 37-1) were the most abundant in all samples. The largest number of *Sulfurovum*-like SP3 sequences were present in the PC08-64 and 66 sites (Figure S6). The abundance of epsilonproteobacterial-affiliated sequences in the metagenomes from S(0)-rich biofilms supports previous observations and further indicates a role for these organisms in the subsurface sulfur cycle.

### Evidence for epsilonproteobacterial sulfide oxidation

Sulfur exists in a broad range of oxidation states (−2 to +6) in the environment, offering the possibility for numerous microbial energy-harvesting transformations and a variety of ecological niches based on differences in energy metabolisms. Lithotrophs capable of oxidizing reduced sulfur compounds include phylogenetically and physiologically diverse bacteria and archaea. These organisms exhibit variations in preferred reduced sulfur substrates, energy conservation strategies, and carbon fixation pathways. The metagenomes we sequenced contained pathways—affiliated largely with members of the Gamma- and Epsilonproteobacteria—for the oxidation of reduced inorganic sulfur species via the sulfur oxidation complex (Sox) and by sulfide-quinone oxidoreductases (Sqr) and flavocytochrome *c* (Fcc).

#### The Sox system

**Figure 2:**
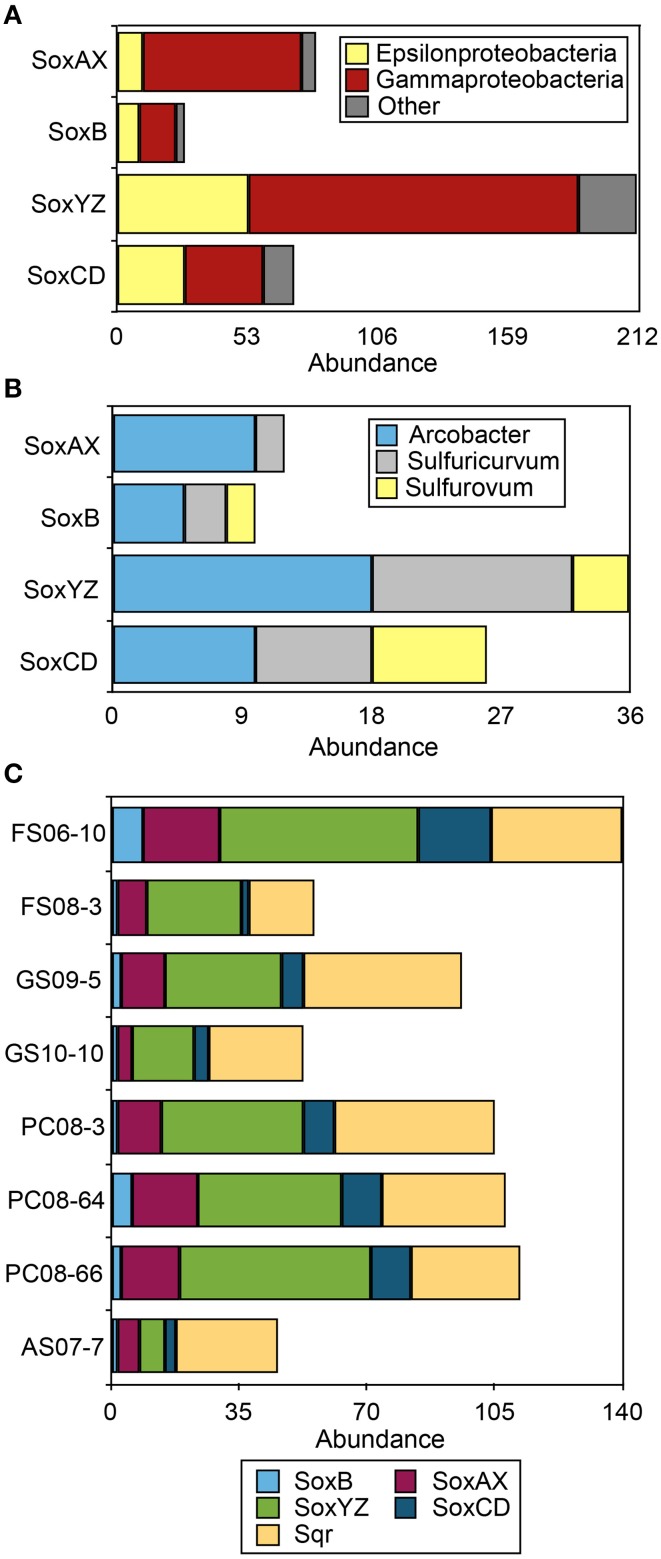
**Gene abundance of Sox and Sqr sequences in the 8 sulfidic cave biofilm metagenomes. (A)** Taxonomic affiliation of Sox sequences in the eight metagenomes at the class level. **(B)** Abundance of epsilonprotebacterial Sox sequences at the genus level. **(C)** Abundance of Sox and Sqr sequences in each metagenome. Sox sequence abundance is given for the protein complexes that are part of the Sox system, SoxB, SoxAX, SoxCD, and SoxYZ. Other indicates all phyla represented by less than 5% of the total sequences in any metagenome.

The Sox system is a widely distributed pathway for the complete oxidation of thiosulfate to sulfate. It includes four central protein components: SoxXA (a heterodimeric *c*-type cytochrome), SoxYZ (a heterodimeric sulfur-binding protein), SoxB (a thiol sulfate esterase), and SoxCD (a sulfur dehydrogenase). Organisms with all four of these complexes are capable of oxidizing thiosulfate and presumably sulfide completely to sulfate (Friedrich et al., 2001). Organisms lacking SoxCD accumulate S(0) as an intermediate and may employ other pathways [i.e., reverse dissimilatory sulfite reductase (rDsr)] to further oxidize S(0) (Dahl et al., 2008; Grein et al., 2010). The Sox multienzyme complex is present in many environmental species of Epsilonproteobacteria including the genomes of *Sulfurovum*, *Nitratiruptor*, *Sulfurimonas*, and *Arcobacter* spp. (Nakagawa et al., 2007; Toh et al., 2011; Grote et al., 2012). Component proteins affiliated with these clades have been recovered from the DNA, mRNA, and protein fractions of number of Epsilonproteobacteria-rich environmental samples (i.e., Handley et al., 2012; Akerman et al., 2013; Headd and Engel, 2013). The majority of Sox sequences recovered from the 8 metagenomes were affiliated with Gammaproteobacteria (Figure 2A; Table S5), even though the Sox multi-enzyme system including SoxB has previously been implicated as a main sulfur-oxidizing pathway in Epsilonproteobacteria (Yamamoto and Takai, 2011). We recovered 29 full-length SoxB sequences from the assembled data, including 9 that were affiliated with Epsilonproteobacteria (Figure S7). Homologs of Sox component proteins affiliated with the Gammaproteobacteria were the most abundant regardless of sample site, followed by sequences affiliated with the Epsilonproteobacteria (Figure 2A). Sox genes affiliated with *Sulfurovum* spp. (i.e., *Sulfurovum* sp. NBC 37-1 and *Sulfurovum* sp. AR) were not abundant despite the abundance of 16S rRNA and ribosomal protein SP3 sequences as well as multiple functional genes (i.e., NapA, AclA, see below) affiliated with this genus (Figure 2B). In all the metagenomes, SoxYZ sequences were the most abundant, followed by SoxAX and SoxCD. No SoxAX sequences affiliated with *Sulfurovum*-like organisms were recovered. The majority of epsilonproteobacterial Sox sequences were affiliated with *Arcobacter* spp. and *Sulfuricurvum* spp. (Figure 2B) suggesting these taxa are capable of complete oxidation of reduced sulfur to sulfate.

Recent combined metagenomic and metaproteomic (proteogenomic) studies of subsurface sediments recovered near complete genomes of *Sulfurovum*-like spp. that lacked the full complement of Sox proteins, but contained the genes for *soxC, D, Y*, and *Z* (Handley et al., 2012). Similarly, in a terrestrial sulfidic spring where epsilonproteobacterial 16S rRNA gene sequences were abundant, no *soxB* genes affiliated with this class were recovered with degenerate primer sets that had previously amplified epsilonproteobacterial *soxB* (Headd and Engel, 2013). These observations highlight the possibility that certain Epsilonproteobacteria may employ incomplete or alternative Sox pathways not yet known from isolates.

#### Sqr and Fcc

The metagenomes contained more than 250 sequences encoding Sqr homologs (Figure 2C) while Fcc homologs were less abundant (*n* = 51). Sqr sequences can be divided into 6 classes, each presumably with a slightly different function (Gregersen et al., 2011). The most abundant forms recovered here, SqrD (*n* = 64) and SqrF (*n* = 58) (Figure S8), are common in Proteobacteria, but have not been biochemically characterized in Epsilonproteobacteria. In the GSB *Chlorobaculum tepidum*, SqrD is responsible for most of the Sqr activity under normal growth conditions while SqrF is important for growth at high sulfide concentration (≥4 mM) (Chan et al., 2008a,b; Holkenbrink et al., 2011). There is only rough correlation between the topology of phylogenetic trees between 16S rRNA sequences and Sqr (and Fcc) sequences from the same organisms (Pham et al., 2008); therefore, the phylogenetic affiliation of Sqr and Fcc homologs are difficult to assign. However, the presence of Sqr in characterized Epsilonproteobacteria that are the closest characterized relatives of the cave spp. (i.e., *Sulfurovum* sp. NBC37-1), coupled to the large number of 16S rRNA sequences in the metagenomes most closely related to members of this genus suggests that a number of the Sqr homologs could be of Epsilonproteobacterial origin.

The abundance of Fcc and Sqr homologs, including all types of Sqrs (as defined in Gregersen et al., 2011), suggests that Epsilonproteobacterial Fcc- and Sqr-catalyzed sulfide oxidation—both of which would result in the formation of S(0)—may contribute to the perennially abundant S(0) in the Frasassi and Acquasanta Terme cave biofilms. Sqr donates electrons from sulfide to the electron transport chain at the level of the quinone pool, while electrons derived from Fcc-catalyzed sulfide oxidation are donated at the level of cytochrome *c*. The energy yield of Sqr-derived electrons should therefore be greater as proton motive force is generated when electrons pass though the cytochrome *b/c*_1_ complex downstream of the quinone pool (Oh-oka and Blankenship, 2004). In purple sulfur bacteria that have SqrD, intracellular sulfur globules are produced, while SqrB-containing strains produce extracellular sulfur. In contrast, all green sulfur bacteria have SqrD/SqrX and produce only extracellular sulfur globules (Gregersen et al., 2011). The most abundant Sqr type observed in the metagenomes, SqrF, is expressed in GSB grown under high sulfide conditions (≥4 mM). In cultures of the GSB *C. tepidum*, sulfide is oxidized to extracellular sulfur globules until sulfide becomes limiting, after which elemental sulfur is utilized as the preferred electron donor (Chan et al., 2008a,b). In samples of sulfur-rich biofilms where Epsilonproteobacteria are abundant (verified with FISH; i.e., Macalady et al., 2008) from Frasassi and Acquasanta Terme, intracellular sulfur globules are not observed. This observation, coupled to the metagenomic data presented here, suggests either rapid cycling of intracellular sulfur or Sqr-catalyzed sulfide oxidation resulting in extracellular deposition of sulfur.

#### Intracellular S(0) oxidation

Consistent with all other available genomic data for members of the Epsilonproteobacteria, no genes encoding the reverse dissimilatory sulfite reductase complex (rDsrAB, encoded by *dsrAB*) were affiliated with this class. The eight metagenomes contain 35 congruent DsrA and B sequences. Of these, 14 group with Dsr genes for dissimilatory sulfate reduction. The others branch with rDsrAB from sulfur-oxidizing Gammaproteobacteria and Chlorobi (Figure S9), in which case they are presumably function in the oxidation of intracellularly stored sulfur (Dahl et al., 2008; Grein et al., 2010). In addition, sequences recovered that encode the sulfur globule proteins SgpA, SgpB, and SgpC [which form a protein envelope around intracellular sulfur globules (Brune, 1995)] were all affiliated with the Gammaproteobacteria.

The presence of genes encoding all of the Sox component proteins and rDSR pathways in the metagenomes suggests that some organisms in the cave biofilms—specifically Gammaproteobacteria and certain spp. of Epsilonproteobacteria including *Arcobacter* spp. and *Sulfuricurvum* spp.—are capable of complete sulfide oxidation to sulfate, but, in the metagenomes, the genetic machinery for these complete sulfide oxidation pathways is relatively rare compared with other sulfur oxidation mechanisms. For example, fewer than 30 copies of SoxCD (necessary for complete oxidation of reduced sulfur to sulfate) were recovered, and 21 copies of rDsr (involved in oxidation of intracellular sulfur to sulfate) in contrast to the greater abundance of copies of Sqr and Fcc (250 and 51 sequences, respectively). Sqr, which catalyzes the oxidation of sulfide to sulfur, is known to play a physiological role in both energy transduction and sulfide detoxification. Regardless of the *in situ* activity of Sqr, our data suggest a prominent role for Sqr and Fcc in biological sulfide oxidation in the cave waters, likely resulting in S(0) formation.

### Epsilonproteobacteria and carbon fixation

Epsilonproteobacteria have been implicated as primary producers in many aphotic, sulfidic environments including deep-sea vents and sulfidic caves and springs (Campbell et al., 2006). All autotrophic Epsilonproteobacteria characterized to date employ the reductive tricarboxylic acid (rTCA) cycle for carbon fixation (Hügler et al., 2011). ATP citrate lyase, comprised of an alpha and a beta subunit—AclA and AclB, respectively—and 2-oxogluterate ferredoxin oxidoreductase are required to catalyze CO_2_ fixation via the rTCA cycle. We recovered 35 full-length copies of AclA, encoded by *aclA*, from the eight metagenomes. The majority of the AclA sequences (*n* = 31) branch within the Epsilonproteobacteria (Figure S10). Genes encoding RuBisCO, the key enzyme in the Calvin-Benson-Bassham (reductive pentose phosphate) cycle, were more abundant than AclA and included 59 copies of *cbbL*, (Form I RuBisCO), and 85 copies of *cbbM* genes (Form II RuBisCO). Many isolate genomes encode both forms of RuBisCO, hindering the interpretation of the abundance of RuBisCO-encoding genes in the metagenomes. Regardless, the high abundance of genes encoding RuBisCO compared to AclA suggests that organisms other than Epsilonproteobacteria also contribute to primary production in the sulfidic streams of the Frasassi and Rio Garaffo cave systems.

### Genetic potential for denitrification by cave epsilonproteobacteria

Most of the Epsilonproteobacteria isolated from deep-sea hydrothermal vents can conserve energy by reducing nitrate, either to ammonia or N_2_ (Campbell et al., 2006). The presence of a highly conserved gene cluster encoding Nap, a high affinity periplasmic nitrate reductase complex, in these vent Epsilonproteobacteria suggests that the use of nitrate as a terminal electron acceptor is widespread in this group (Vetriani et al., 2014). Nap has a higher affinity for nitrate compared to the membrane-bound form of nitrate reductase (Nar) and is typically expressed under anoxic or micro-oxic conditions. Although the physiological function of Nap may differ among bacteria, it is expressed during anaerobic growth of *Wolinella succinogenes*, catalyzing the first step of nitrate reduction to ammonia (Grove et al., 1996; Simon et al., 2003) and is common in the genomes of diverse Epsilonproteobacteria. Deep-sea mesophilic species tend to reduce nitrate to N_2_, presumably via NapA, while thermophilic species tend to carry out ammonification through the activity of NapA and Nrf (Kern and Simon, 2009; Vetriani et al., 2014). Lower nitrate concentrations in thermal fluids are postulated to play a role in selecting for ammonification as the more common pathway at higher temperatures (Blöchl et al., 1997; Vetriani et al., 2004; Perez-Rodriguez et al., 2012); however, this hypothesis has not been thoroughly tested.

**Figure 3:**
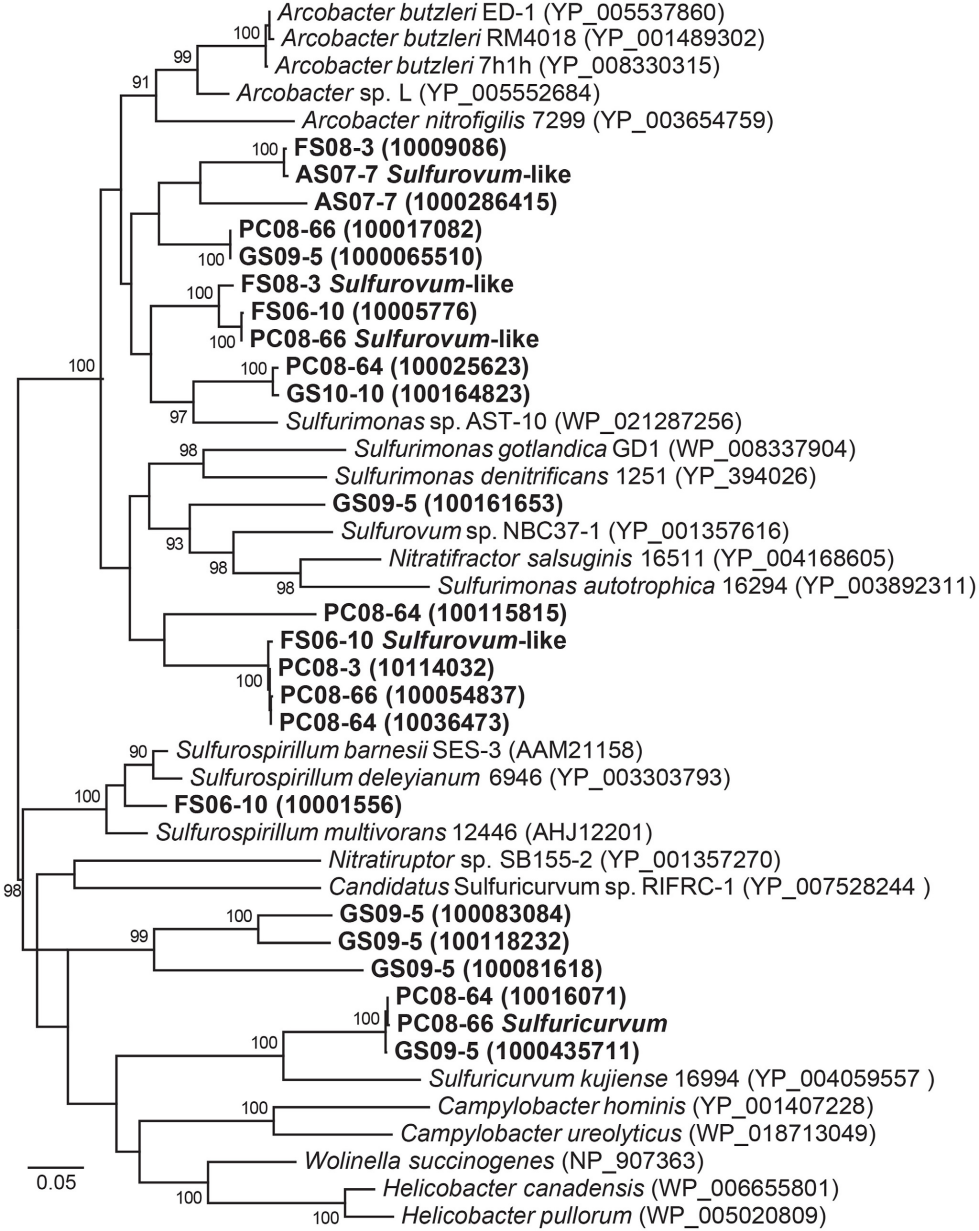
**Maximum likelihood phylogeny of NapA sequences affiliated with Epsilonproteobacteria from all the metagenomes and sequences mined from complete and draft genomes available from NCBI and IMG/M**. Accession numbers or IMG designations are given in parentheses. Bootstrap support values >90 based on 1000 bootstrap samplings are shown for each node.

The eight metagenomes contain more than 20 NapA sequences, and all of these sequences cluster with Epsilonproteobacteria (Figure 3) despite the observation that nitrate levels are consistently below detection limits (~0.7 μM) in both the mesophilic Frasassi and thermal Rio Garrafo cave waters. If denitrification is occurring, nitrate must be rapidly scavenged and cycled, perhaps facilitated by the expression of high affinity Nap. Alternatively, nitrate may be periodically available in specific microbial niches. Similar to the rapid cycling of sulfur species observed in oxygen-free waters off the Chilean coast (Canfield et al., 2010), a cryptic N cycle linked to sulfide oxidation and carbon fixation could be operating in the cave streams, therefore affecting biogeochemical cycling in the subsurface.

### Epsilonproteobacteria genomic bins

We retrieved five nearly-complete epsilonproteobacterial genomes from AS07-1, FS06-10, FS08-3 and PC08-66 after using tetranucleotide frequency to define genomic bins from the metagenomic data.

#### Sulfurovum-like genomes

**Figure 4:**
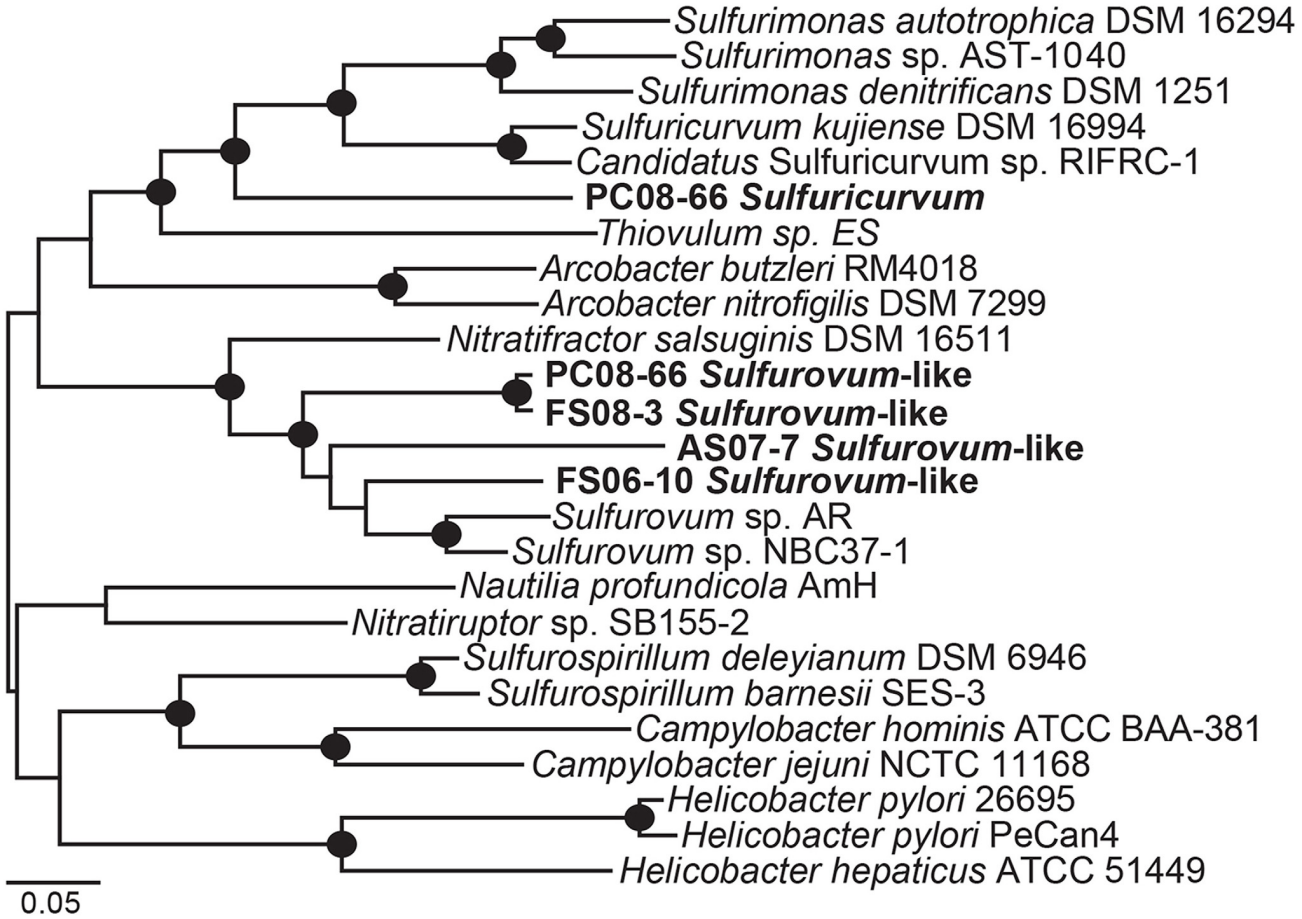
**Maximum likelihood phylogenetic tree of 19 concatenated single-copy ribosomal proteins (Table S2) showing the taxonomic placement of the Epsilonproteobacteria genomic bins**. Black circles indicate bootstrap support values >90 based on 1000 bootstrap samplings.

**Figure 5:**
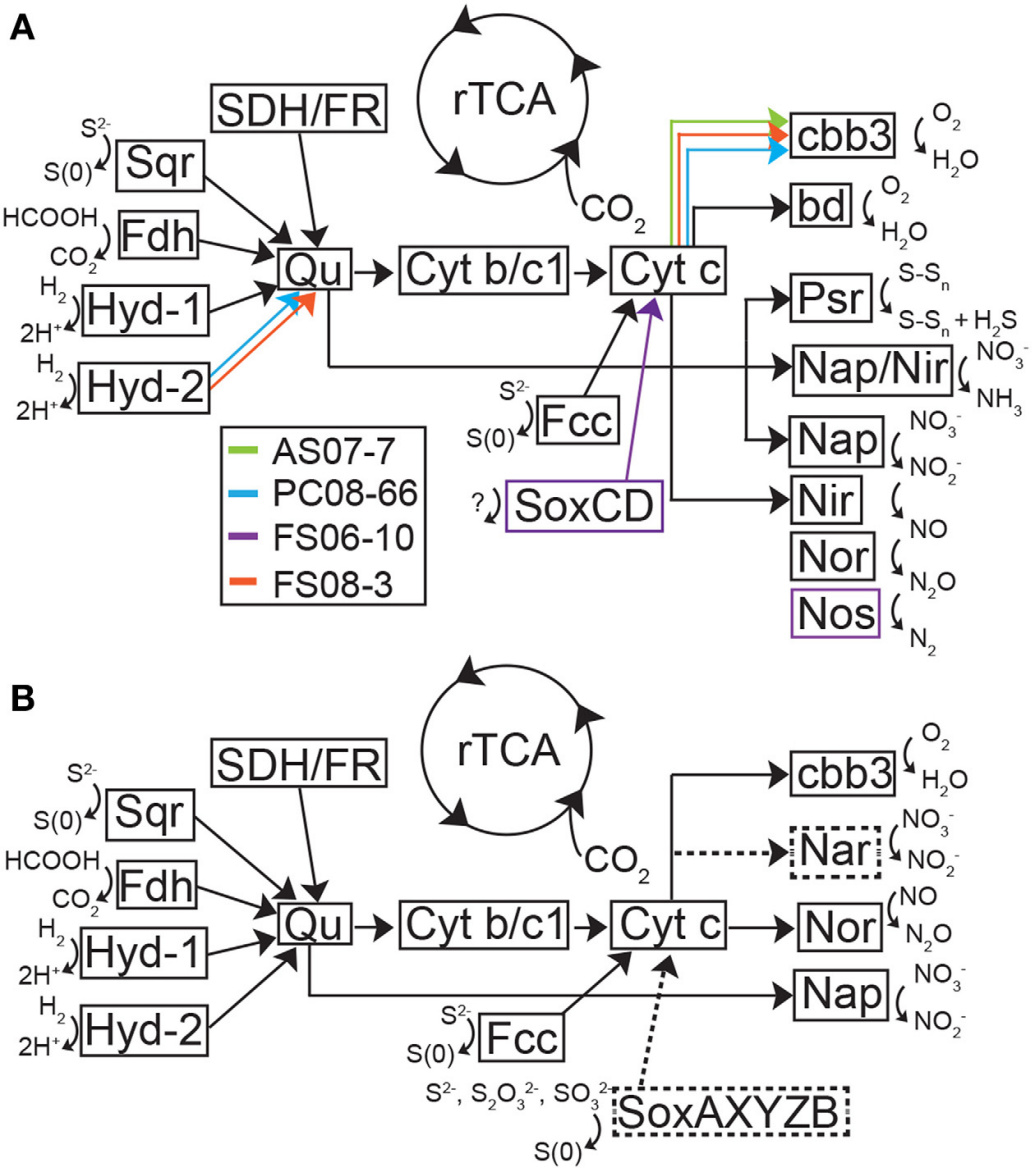
**Theoretical model of metabolic processes and electron transport in the *Sulfurovum*-like genomic bins (A) and the *Sulfuricurvum* genomic bin (B) inferred from the presence of functional genes in these bins. (A)** Model of metabolic pathways and electron transport in the *Sulfurovum*-like genomes based on putative proteins encoded in the genome bins. Black arrows represent genes present in all organisms in the genome and colored arrows represent functional genes identified in a subset of the four genomes. **(B)** Model of metabolic pathways and electron transport in the *Sulfuricurvum* genome based on putative proteins encoded in the genome bin. Dashed lines and boxes represent enzymes and reactions not present in the *Sulfurovum-like* genomes. Both models were based on Grote et al. (2012). bd, *bd*-type quinol oxidase; cbb3, cytochrome c oxidase; Cyt *b*/*c*1, quinone cytochrome oxidoreductase; Fdh, formate dehydrogenase; Hyd, hydrogenase; Nap, nitrate reductase; Nar, nitrate reductase, Nir, nitrite reductase; Nor, nitric oxide reductase; Nos, nitrous oxide reductase; Psr, polysulfide reductase; Qu, quinone; SDH/FR, succinate dehydrogenase/fumarate reductase; Sox, sulfur oxidation complex; Sqr, sulfide-quinone oxidoreductase.

Phylogenetic analysis of 19 concatenated phylogenetic marker genes (Table S2) indicates that four of the Epsilonproteobacteria genomes are most closely related to *Sulfurovum* sp. NBC37-1 and *Sulfurovum* sp. AR (Figure 4). The genomic bins did not contain 16S rRNA sequences; however, based on the 19-ribosomal-protein concatenated alignment the genomic bins represent *Sulfurovum*-like organisms. The four draft genomes are 90–95% complete, ranging in size from 2.1 to 2.7 Mbp with average GC contents ranging from 33.9 to 41.2% (Table 2) based on the presence of conserved single-copy marker genes (Table S1). These genome sizes are consistent with those of the cultivated *Sulfurovum* strains *Sulfurovum* sp. NBC37-1 and *Nitratiruptor* sp. SB155-2, which contain ~2.5 Mbp and 43.2% GC and ~1.8 Mbp and 39.7% GC, respectively (Nakagawa et al., 2007). Strains NBC37-1 and SB155-2 were both isolated from deep-sea hydrothermal vents and are lithoautotrophs capable of oxidizing hydrogen or sulfur compounds under microaerophilic or anaerobic conditions (Nakagawa et al., 2005b). The metabolic potential encoded in the *Sulfurovum*-like genomic bins was similar regardless of both geographic location and sample site geochemistry (Table 1). The four cave *Sulfurovum*-like genomic bins contain have strong similarities with these cultivated strains in terms of metabolic pathways and electron transport (Figure 5A) and similar to *Sulfurovum* sp. NBC37-1 and *Nitratiruptor* sp. SB155-2 and all known epsilonproteobacterial autotrophs (Hügler et al., 2011), each cave *Sulfurovum*-like genomic bin encodes all of the genes necessary for CO_2_ fixation via the reductive tricarboxylic acid cycle (rTCA) (Figure 5A).

**Table 2:** **Statistics for the *Sulfurovum*-like and *Sulfuricurvum* genomic bins**.

**Genomic bin**	**% GC**	**Total bp**	**Number of scaffolds**	**Longest scaffold (bp)**	**N50 (bp)**	**Genes**	**% complete**
AS07-7 *Sulfurovum*-like	33.9	2744436	79	316531	50837	2949	95.0
PC08-66 *Sulfuricurvum*	46.9	2292648	20	489506	256334	2318	95.0
PC08-66 *Sulfurovum*-like	41.2	2111759	120	437665	63533	2413	95.0
FS06-10 *Sulfurovum*-like	35.3	2643792	122	361107	37111	2706	90.0
FS08-3 *Sulfurovum*-like	39.7	2543982	68	427958	70890	2662	95.0

Sample designations are given in Table 1.

The *Sulfurovum*-like genomic bins encode the complete pathway for oxidative phosphorylation along with high affinity O_2_ terminal oxidases indicating the organisms are capable of using oxygen as a terminal electron acceptor. Both FS genomes and the AS genome encode *cbb3*-type cytochrome-*c* oxidases while the PC genome only encodes a *bd*-type quinol oxidase (Figure 5A). The AS genome also contains the genes for a *bd*-type quinol oxidase (Figure 5A). Both oxidases are high-affinity terminal oxygen reductases capable of functioning under low oxygen concentrations (Pitcher and Watmough, 2004; Borisov et al., 2011). An alternative function of *cbb3*-type cytochrome-*c* oxidases in catalytic NO reduction has been suggested in *Sulfurospirillum denitrificans*, but this activity has not been demonstrated (Sievert et al., 2008). All of the *Sulfurovum*-like genomes encode a type-1 NADH dehydrogenase (Nuo), a formate dehydrogenase (FdhABC), cyctochrome *c*_553_, and multiple [NiFe]-hydrogenases (including cytoplasmic and membrane-bound forms), suggesting they can use H_2_ and formate as electron donors (Figure 5A). All four genomic bins also encode the enzymes necessary for assimilatory nitrate reduction (NasAB/NarB and NirA); ammonium transport (Amt), and Mo-dependent nitrogenases that catalyze dinitrogen reduction (Table 3).

**Table 3:** **Key genes encoding respiratory complexes and proteins involved in nitrogen and sulfur cycling identified in the genomic bins^a,b^**.

**NITROGEN CYCLE**													
**Genomic Bin**	**Cyt**	**Sdh**	**Nap**	**Nas^c^**	**Nif^d^**	**Nir^e^**	***c*Nor^f^**	**NosZ**	**Nrf**		
AS07-7 *Sulfurovum*-like	*cbb*_3_, *bd*	✓	✓	✓	✓	✓	✓	✓	—		
PC08-66 *Sulfuricurvum*	*cbb*_3_	✓	✓	✓	✓	—	✓	✓	—		
PC08-66 *Sulfurovum*-like	*bd*	✓	✓	✓	✓	✓	✓	✓	—		
FS06-10 *Sulfurovum*-like	*cbb*_3_	✓	✓	✓	✓	✓	✓	✓	✓		
FS08-3 *Sulfurovum*-like	*cbb*_3_	✓	✓	✓	✓	✓	✓	✓	—		
**SULFUR CYCLE**													
**Genomic Bin**	**Sox^g^**	**Sqr^h^**	**Fcc**	**Psr**	**Rhd**	**Sat**	**Sde**	**Sir**	**Sor**	**Sud**	**Ttr**
AS07-7 *Sulfurovum*-like	—	2	✓	✓	✓	✓	✓	✓	—	—	✓
PC08-66 *Sulfuricurvum*	BAXYZ	3	✓	✓	✓	✓	✓	—	—	—	✓
PC08-66 *Sulfurovum*-like	—	2	✓	✓	✓	✓	✓	—	—	—	✓
FS06-10 *Sulfurovum*-like	CD	2	✓	✓	✓	✓	✓	—	✓	✓	✓
FS08-3 *Sulfurovum*-like	—	1	✓	✓	✓	✓	✓	✓	—	—	✓

a ✓indicates genes were present,—indicates genes were not identified.

b Cyt, terminal respiratory complex; Rhd, rhodanese; Sde, thiosulfate reductase; Ttr, tetrathionite reductase.

c Assmilatory nitrate reduction (genes encoding NasAB/NarB, NirA).

d Presence of genes encoding nitrogenase structural proteins NifH, D, and K.

e Cytochrome cd_1_ (genes encoding NirS).

f cNOR, cytochome c:nitric oxide reductase (genes encoding NorCB complex).

g Component proteins identified are indicated.

h Numbers indicate unique Sqr sequences identified in each genomic bin.

The *Sulfurovum*-like genomes encode a periplasmic nitrate reductase complex (Nap; encoded by *nap*) and a putative assimilatory nitrate reductase (NarB; encoded by *narB*) (Table 3). The FS06 genomic bin also encodes a periplasmic cytochrome *c* nitrite reductase (Nrf) (Figure 5A). Most of the ammonifying Epsilonproteobacteria characterized to date use Nrf to reduce nitrite to ammonium (Kern and Simon, 2009). In addition to nitrite, Nrf also acts on other substrates such as nitric oxide, H_2_O_2_, and sulfite. All of the genomic bins encode the homologs of the enzymes necessary for complete reduction of nitrate to N_2_, including the periplasmic nitrate reductase (Nap), cytochrome *cd*_1_ nitrite reductase (NirS) a nitric oxide reductase (Nor), and nitrous oxide reductase (NosZ).

The *Sulfurovum*-like genomes encode multiple homologs of sulfide-quinone oxidoreductase (Sqr), and flavocytochrome *c* (Fcc), a sulfide:cytochrome *c* oxidoreductase (Figure 5A). The genome of *Sulfurovum* sp. NBC37-1 encodes two Sqr homologs that have been indirectly implicated in filamentous S(0) formation by deep-sea vent Epsilonproteobacteria (Taylor and Wirsen, 1997; Nakagawa et al., 2007). However, the activity of the Sqr homologs has not been examined in pure culture or *in situ*. Multiple forms of Sqr are present in the bins, including homologs of SqrB, SqrD, and SqrF. The presence of genes for nitrate respiration, either to ammonium or complete denitrification to N_2_, together with the presence of *sqr* genes, suggests that the *Sulfurovum*-like populations are capable of nitrate-dependent sulfide oxidation (Simon and Klotz, 2013). Although the FS06 genomic bin encodes SoxCD, all of the genomic bins lack Sox component proteins AX, B, and YZ (Figure 5A; Table 3). In order to insure that possible epsilonproteobacterial *sox* genes were not excluded by our binning approach (e.g., due to horizontal gene transfer events), we manually inspected all scaffolds encoding *sox* genes for %GC, tetranucleotide frequency, genomic context and best BLASTP/X hits of all genes to verify that the *sox*-containing contigs were correctly binned. Using this approach, we were unable to assign any more sequences to the *Sulfurovum*-like genomic bins. All of the bins encode additional enzymes involved in sulfur transformations, including sulfate adenylyltransferase (Sat); a thiosulfate reductase complex (Sde, encoded by *sde*) that mediates the reduction of thiosulfate to sulfide (Lohmayer et al., 2014); polysulfide reductase (Psr, encoded by *psr*); tetrathionate reductase (Ttr); and rhodanese, which catalyzes the oxidation of thiosulfate to sulfite. The FS06 genomic bin encodes sulfur oxygenase reductase (Sor), an enzyme that catalyzes the disproportionation of S(0) to sulfite, sulfide, or thiosulfate; and Sud, a periplasmic sulfide dehydrogenase. The AS and FS08 genomic bins encode sulfite reductase (Sir), which catalyzes the reduction of sulfite to hydrogen sulfide.

#### Ecological success of sulfurovum-like populations

The nearly-complete *Sulfurovum*-like genomes we recovered were similar to each other in that they encode genes necessary for oxygen or nitrate-dependent autotrophic growth on reduced sulfur via the rTCA cycle. These organisms appear to be genetically poised to thrive in the high-sulfide, low-oxygen niche of fast flowing cave waters, while more subtle variations in genetic potential, particularly those involved in redox processes or oxygen-tolerance, may facilitate fine-scale niche partitioning among the cave populations. The *Sulfurovum*-like genomes encode high affinity oxidases (*cbb3*-type or *bd*-type quinone-type) and periplasmic nitrate reductases (Nap) (Figure 5A). The high affinity oxidases can operate even at low oxygen concentrations while Nap is functional in some organisms under mico-oxic conditions (Moreno-Vivian et al., 1999). If Nap in the *Sulfurovum*-like genomes is oxygen-tolerant, both nitrate and oxygen could serve as efficient electron acceptors in these dynamic environments, even in the presence of low oxygen or nitrate concentrations. The FS06 genomic bin encodes both Nap and Nrf, which are employed by ammonifying Epsilonproteobacteria (Kern and Simon, 2009). This is the least energetically favorable sulfide oxidation pathway for S(0) production (Equation 6) but also requires less nitrate than both complete or incomplete sulfide oxidation via denitrification. In tropical sediments, the combination of low nitrate and low organic matter at elevated temperatures tends to favor nitrate reduction to ammonium (Dalsgaard and Thamdrup, 2002; Rysgaard et al., 2004; Dong et al., 2011) while sulfide concentrations above 0.3 mM may inhibit denitrification (Knowles, 1982). However, the fate of nitrate when nitrate reduction is driven by autotrophic sulfide oxidation may vary according to the taxonomy of sulfide oxidizers (Sayama et al., 2005). In the Frasassi and Acquasanta Terme caves, the ammonia-rich waters are deficient in nitrate, conditions that could reflect incomplete sulfide oxidation coupled with nitrate reduction.

Although poorly understood, the presence and putative expression of different Sqr homologs may also serve as a selective advantage to sulfur oxidizing organisms in dynamic environments where sulfide (and oxygen) concentrations fluctuate with daily and seasonal variations in water level and flow. All of the *Sulfurovum*-like genome bins also encode a polysulfide reductase, Psr, suggesting they are capable of using H_2_ or formate as an electron donor and polysulfide as an electron acceptor. The AS and FS06-10 genomes encode group 1 [NiFe] enzymes that are homologous to the hydrogen uptake enzyme Hyd-1 in *Escherichia coli*. The PC and FS08-3 genomes contain homologs of both Hyd-1 and Hyd-2. In *E. coli*, the hydrogen uptake enzymes are expressed at different phases and exhibit stark differences in activity—Hyd-1 is an oxygen-tolerant form that catalyzes H_2_ oxidation under oxidizing conditions and Hyd-2 is an anaerobic quinone-reactive uptake hydrogenase (Hyd-2) that is efficient under reducing conditions (Lukey et al., 2010). Although sulfide does not appear to be limiting, fluctuations in stream flow may dictate nitrate, oxygen, and sulfide levels, conferring a selective advantage for organisms capable of polysulfide respiration in the sulfur-rich biofilms when more desirable electron donors are not available. However, polysulfide respiration is far less energetically favorable (~30 kJ mol^−1^ H_2_ or formate) than sulfide oxidation and thus presumably not the favored metabolism of the cave Epsilonproteobacteria. Mixotrophic and heterotrophic growth has been observed in other Epsilonproteobacteria (Campbell et al., 2006) and could also be an important metabolic strategy for the cave *Sulfurovum*-like populations.

The FS06-10 genome encodes several additional enzymes which mediate sulfur cycling, including a sulfur oxygenase reductase (Sor) which catalyzes the oxygen-dependent disproportionation of sulfur, producing sulfite, thiosulfate, and sulfide and a periplamsic sulfide dehydrogenase (Sud) which may enhance the activity of Psr. Sor is not widespread in nature and the activity of Sor in sulfide oxidizers has mainly been characterized in thermoacidophilic archaea and hypthermophilic bacteria (Veith et al., 2011). Further analysis will be necessary to determine the role of Sor *in situ* in the FS06-10 *Sulfurovum*-like population. The presence of genes encoding Psr, Sud, and Sor suggests that both oxygen-dependent sulfur disproportionation and efficient anaerobic polysulfide respiration may be an important advantage for the *Sulfurovum-like* population in this niche.

#### Sulfuricurvum genome

A nearly complete genome (>95%) of a *Sulfuricurvum*-like sp. was recovered from the PC08-66 biofilm sample. The draft genome contains 2.3 Mbp over 20 scaffolds with an average GC content of 46.9% (Table 2). Phylogenetic marker genes indicate that the genome is within the *Sulfuricurvum* genus, which contains a single cultured representative, *Sulfuricurvum kujiense* (Figure 4) isolated from an underground crude-oil storage cavity (Kodama and Watanabe, 2003, 2004). 16S rRNA sequence analysis (Figure S11) indicates that the cave *Sulfuricurvum* sp. is distantly related to *S. kujiense* as well as to *Candidatus* Sulfuricurvum sp. RIFRC-1, an uncultivated Epsilonprotebacteria abundant in the weakly sulfidic freshwater Rifle aquifer in Colorado (Handley et al., 2014). *S. kujiense* is a lithoautotroph that can use sulfide, S(0), thiosulfate or hydrogen as an electron donor and nitrate or low concentrations of oxygen as an electron acceptor (Kodama and Watanabe, 2003, 2004). The PC *Sulfuricurvum* sp. encodes all the enzymes necessary for autotrophic growth via the rTCA cycle, a *cbb3*-type cytochrome *c* oxidase, and a Type-1 NADH dehydrogenase (encoded by *nuo*). Like *S. kujiense* and *Ca.* Sulfuricurvum sp. RIFRC-1 (Han et al., 2012; Handley et al., 2014), the cave *Sulfuricurvum* genome contains genes for multiple [NiFe]-hydrogenases including two membrane-bound Group 1 forms (Figure 5B). The presence of Hyd-1 and Hyd-2 membrane-bound forms indicate that this organism is capable of H_2_ oxidation under varying redox conditions (Lukey et al., 2010). Like the genomes of *S. kujiense* and *Ca.* Sulfuricurvum sp. RIFRC-1, the PC *Sulfuricurvum* bin also encodes a periplasmic nitrate reductase (Nap), a membrane-bound nitrate reductase (Nar), and a nitric oxide reductase (Nor) (Table 3; Figure 5B). The genome also encodes an ammonium transporter (Amt), and a molybdenum-dependent nitrogenase.

Similar to the *Sulfurovum*-like genomes, the PC *Sulfuricurvum* genome encodes multiple Sqr homologs and an Fcc homolog as well as sulfate adenylyltransferase (Sat), polysulfide reductase (Psr), rhodanese, thiosulfate reductase (Sde), and tetrathionate reductase (Ttr) (Table 3). In addition, the genome encodes the Sox component proteins, SoxAXBYZ, necessary for thiosulfate oxidation (Figure 5B). The genome, although not complete, does not contain *soxCD* genes. *S. kujiense* and *Ca.* Sulfuricurvum sp. RIFRC-1 also lack *soxCD* (Han et al., 2012; Handley et al., 2014). SoxCD, which functions as sulfane dehydrogenase, catalyzes a 6 electron transfer reaction and is required, along with SoxAXBYZ, for the complete oxidation of one molecule of thiosulfate into two molecules of sulfate (Friedrich et al., 2000).

Collectively, the metabolic potential encoded in the *Sulfurovum*-like and *Sulfuricurvum* genome bins indicates these organisms are capable of primary production via the rTCA cycle and can contribute to the sulfur cycle under aerobic, micro-oxic, or anaerobic conditions. However, the lack of genes encoding Sox component proteins in cave *Sulfurovum*-like genomes from several geochemically and geographically distinct sites (Table 1) coupled to the presence of only SoxAX, YZ, and B component proteins in the cave *Sulfuricurvum* suggests that these populations are not capable of complete sulfide oxidation—via Sox, Sqr or Fcc—to sulfate, and thus they may be important producers of S(0) in the subsurface.

## Conclusion

Our data underscore the biogeochemical importance and ecological success of *Sulfurovum*-like Epsilonproteobacteria in sulfide-rich subsurface environments, and suggest that further efforts to obtain cultured representatives are warranted in order to understand their physiology, function and role in S(0) production and biogeochemical cycling in the subsurface as well as in other environments. While the mechanism of extracellular S(0) accumulation remains unknown, S(0) formation would be favored as the major end product of sulfide oxidation in nitrate- and oxygen-limited cave waters where *Sulfurovum*-like Epsilonproteobacteria have been shown to dominate. Our data indicate that abundant cave *Sulfurovum*-like populations are genetically equipped to catalyze S(0) precipitation using either O_2_ or nitrate as a terminal electron acceptor while employing a lithoautotrophic lifestyle, highlighting a likely integral role of S(0) formation in the carbon, nitrogen, and sulfur cycles in the subsurface and in other past and present sulfidic environments where electron acceptors are limiting.

### Conflict of interest statement

The authors declare that the research was conducted in the absence of any commercial or financial relationships that could be construed as a potential conflict of interest.
