# Supplementary material for: Metagenomic insights into S(0) precipitation in a terrestrial subsurface lithoautotrophic ecosystem
Source: Front Microbiol. 2015 Jan 8;5:756. doi: 10.3389/fmicb.2014.00756 (PMC4288042; doi:10.3389/fmicb.2014.00756)
Supplement: Supplementary file 1 [file DataSheet1.PDF]

Table S1. Marker genes used to evaluate genome completion of the Epsilonproteobacteria genome bins. Marker gene abundance is given for all genes except tRNA synthetase genes which are noted as present (Y) or absent (N).

	Genomic Bins				
**Marker Genes**	AS07-7. *Sulfurovum*-like	PC08-66 *Sulfuricurvum*	PC08-66 *Sulfurovum*-like	FS06-10 *Sulfurovum*-like	FS08-3 *Sulfurovum*-like
RecA	1	1	1	1	1
DNA gyrase A	1	1	1	1	1
DNA gyrase B	1	1	1	1	1
RpoD	1	1	1	1	1
SecG	1	1	1	1	1
DNA RNA polymerase, beta subunit	1	1	1	1	1
eolongation factor P	1	1	1	1	1
ribonuclease P	1	1	1	1	1
**Ribsosomal Proteins**					
L1	1	1	1	1	1
L2	1	1	1	1	1
L3	1	1	1	1	1
L4	1	1	1	1	1
L5	1	1	1	1	1
L6	1	1	1	1	1
L7/L12	1	1	1	1	1
L10	1	1	1	1	1
L11	1	1	1	1	1
L13	1	1	1	1	1
L14	1	1	1	1	1
L15	1	1	1	1	1
L16	1	1	1	1	1
L17	1	1	1	1	1
L18	1	1	1	1	1
L19	2	1	1	1	1
L20	1	1	1	0	1
L21	1	1	1	1	1
L22	1	1	1	1	1
L23	1	1	1	1	1
L24	1	1	1	1	1
L25	2	1	1	1	1
L27	1	1	1	1	1
L28	1	0	1	0	1
L29	1	1	1	1	1

**Ribsosomal Proteins**					
S1	1	1	1	1	1
S2	1	1	1	1	1
S3	1	1	1	1	1
S4	1	1	1	1	1
S5	1	1	1	1	1
S6	1	1	1	1	1
S7	1	1	1	0	1
S8	1	1	1	1	1
S9	1	1	1	0	1
S10	1	1	1	1	1
S11	1	1	1	1	1
S12	1	1	2	1	1
S13	1	1	1	1	1
S14	0	0	1	0	1
S15	1	1	1	1	1
S16	2	1	1	1	1
S17	1	1	1	1	1
S18	2	0	1	1	1
S19	1	1	1	1	1
S20	1	1	0	1	1
**rRNA**					
5S rRNA	0	1	0	0	0
16S rRNA	0	0	0	0	0
23S rRNA	0	0	0	0	0
**tRNA synthetase**					
ala	Y	Y	Y	Y	Y
arg	Y	Y	Y	Y	Y
asp	Y	Y	Y	Y	Y
asn	Y	Y	Y	Y	Y
cys	Y	Y	Y	Y	Y
gln	Y	Y	Y	Y	Y
glu	Y	Y	Y	Y	Y
gly	Y	Y	Y	Y	Y
his	Y	Y	N	Y	Y
ile	Y	Y	Y	Y	Y
leu	Y	Y	Y	Y	Y
lys	Y	Y	Y	Y	Y
met	Y	Y	Y	Y	Y
phe	Y	Y	Y	Y	Y
pro	Y	Y	Y	Y	Y
ser	Y	Y	Y	Y	Y

**tRNA synthetase**					
thr	Y	Y	Y	Y	Y
trp	Y	Y	Y	Y	Y
tyr	Y	Y	Y	Y	Y
val	Y	Y	Y	Y	Y

Table S2. Single-copy phylogenetic marker genes used for taxonomic assignment of the epsilonproteobacteria genome bins.

**Ribosomal Protein**
LSU ribosomal protein L15p (L27Ae)
SSU ribosomal protein S5p (S2e)
LSU ribosomal protein L18p (L5e)
LSU ribosomal protein L6p (L9e)
SSU ribosomal protein S8p (S15Ae)
LSU ribosomal protein L5p (L11e)
LSU ribosomal protein L24p (L26e)
LSU ribosomal protein L14p (L23e)
SSU ribosomal protein S17p (S11e)
LSU ribosomal protein L29p (L35e)
LSU ribosomal protein L16p (L10e)
SSU ribosomal protein S3p (S3e)
LSU ribosomal protein L22p (L17e)
SSU ribosomal protein S19p (S15e)
LSU ribosomal protein L2p (L8e)
LSU ribosomal protein L23p (L23Ae)
LSU ribosomal protein L4p (L1e)
LSU ribosomal protein L3p (L3e)
SSU ribosomal protein S10p (S20e)

Table S3. Access to the metagenomes on the Integrated Microbial Genome (IMG-M) site.

Metagenome	Sample ID	Link to IMG data
4094966	AS07_7	https://img.jgi.doe.gov/cgi-bin/mer/main.cgi?section=TaxonDetail&taxon_oid=3300000227
4094997	FS06_10	https://img.jgi.doe.gov/cgi-bin/mer/main.cgi?section=TaxonDetail&taxon_oid=3300000233
4095000	FS08_3	https://img.jgi.doe.gov/cgi-bin/mer/main.cgi?section=TaxonDetail&taxon_oid=3300000236
4094998	GS09_5	https://img.jgi.doe.gov/cgi-bin/mer/main.cgi?section=TaxonDetail&taxon_oid=3300000234
4094994	GS10_10	https://img.jgi.doe.gov/cgi-bin/mer/main.cgi?section=TaxonDetail&taxon_oid=3300000230
4094999	PC08_3	https://img.jgi.doe.gov/cgi-bin/mer/main.cgi?section=TaxonDetail&taxon_oid=3300000235
4094996	PC08_64	https://img.jgi.doe.gov/cgi-bin/mer/main.cgi?section=TaxonDetail&taxon_oid=3300000232
4094984	PC08_66	https://img.jgi.doe.gov/cgi-bin/mer/main.cgi?section=TaxonDetail&taxon_oid=3300000228

[a] Sample IDs are defined in Table S1.

Table S4. Metagenome summary statistics.

Sample ID	Location[a]	Assembled Size (bp)	GC % (Assembled)	Number of sequences	N50	Average read length (>800 bp)
AS07_7	Acquasanta Terme	300,437,395	44.5	293,802	2678	3348
FS06_10	Fissure Spring	242,610,534	44.3	268,733	1770	2668
FS08_3	Fissure Spring	181,303,868	44.5	261,777	1001	2896
GS09_5	Grotto Sulfurea	539,638,524	37.5	532,818	2628	3339
GS10_10	Grotto Sulfurea	581,077,747	39.1	770,535	1106	2255
PC08_3	Pozzo del Cristalli	252,007,248	38.0	293,864	1635	2913
PC08_64	Pozzo del Cristalli	448,373,019	42.7	617,664	1239	2588
PC08_66	Pozzo del Cristalli	270,071,044	43.6	353,471	1078	2298

[a] Acquastana Terme sample from Grotta Nuova di Rio Garrafo.

Table S5. Gene abundance of Sox sequences in the 8 sulfidic cave biofilm metagenomes at the class level.

	FS06-10				FS08-3				GS09-5				GS10-10			
Sox compnent	B	AX	YZ	CD	B	AX	YZ	CD	B	AX	YZ	CD	B	AX	YZ	CD
Epsilonproteobacteria	2	1	8	6	-	1	6	1	2	0	8	4	2	1	8	2
Gammaproteobacteria	5	10	19	10	2	4	18	4	1	6	16	2	1	4	15	2
Other	-	-	6	-	2	5	2	-	-	-	3	-	-	-	2	-
	PC08-3				PC08-64				PC08-66				AS07-7			
Sox compnent	B	AX	YZ	CD	B	AX	YZ	CD	B	AX	YZ	CD	B	AX	YZ	CD
Epsilonproteobacteria	1	2	9	4	1	4	8	5	2	2	8	2	-	-	6	2
Gammaproteobacteria	1	3	18	3	4	8	21	4	1	8	18	4	-	4	9	4
Other	-	-	1	-	-	-	2	-	-	-	1	-	-	-	2	-

Figure S1. Epsilonproteobacteria-rich biofilm from the Frasassi cave system. Stream reach in A is approximately 5 m long. Scale bar in C is 5 microns.

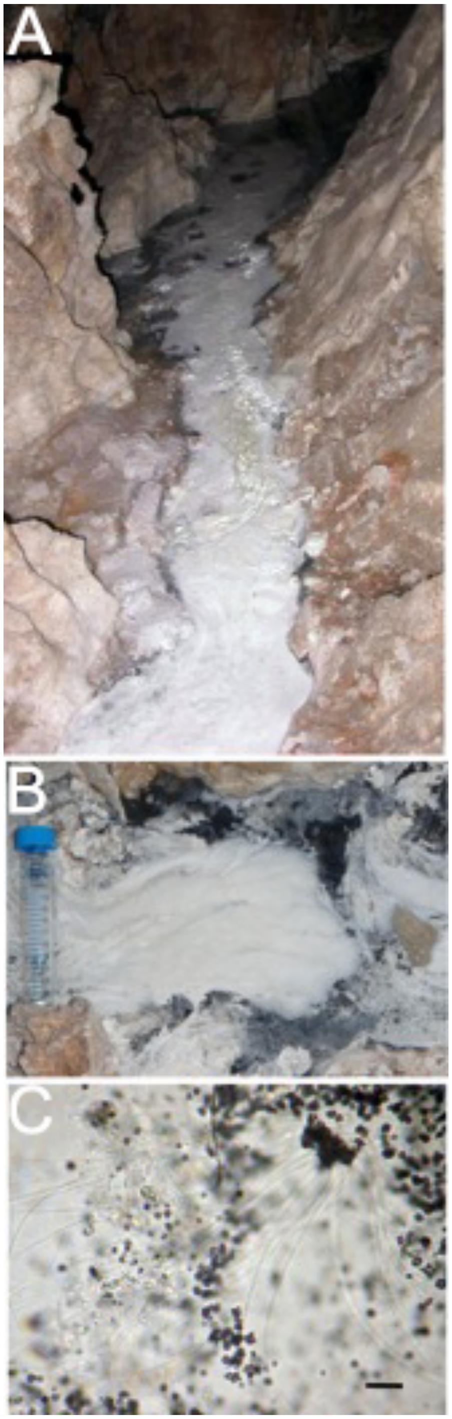

Figure S2. Total sulfur content of Frasassi biofilms and sediment samples collected at the same locations. Error bars represent one standard deviation among samples.

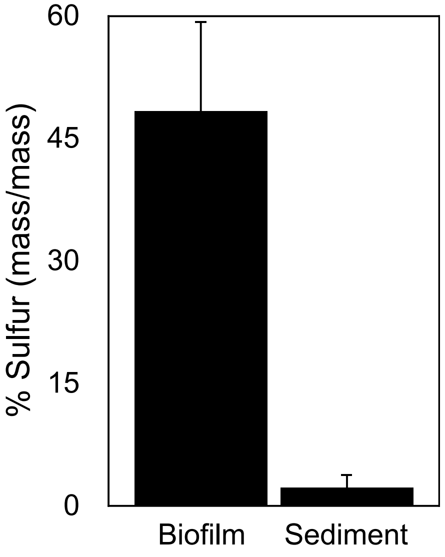

Figure S3. Taxonomic affiliation of all assembled metagenomic sequences at the kingdom level. Sequences affiliated with each kingdom were normalized to the total sequences in each metagenome. Sample designations are given in Table 1.

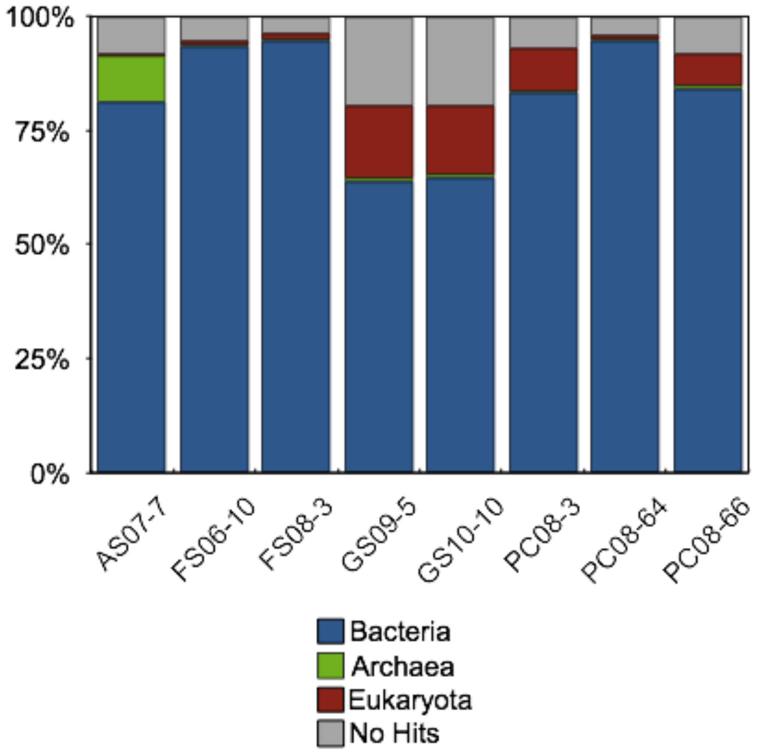

Figure S4. Taxonomic affiliation of bacterial sequences in the eight metagenomes assigned at the phylum-level (except the Proteobacteria which are represented by class). Other indicates all phyla represented by less than 5% of the total sequences in any metagenome.

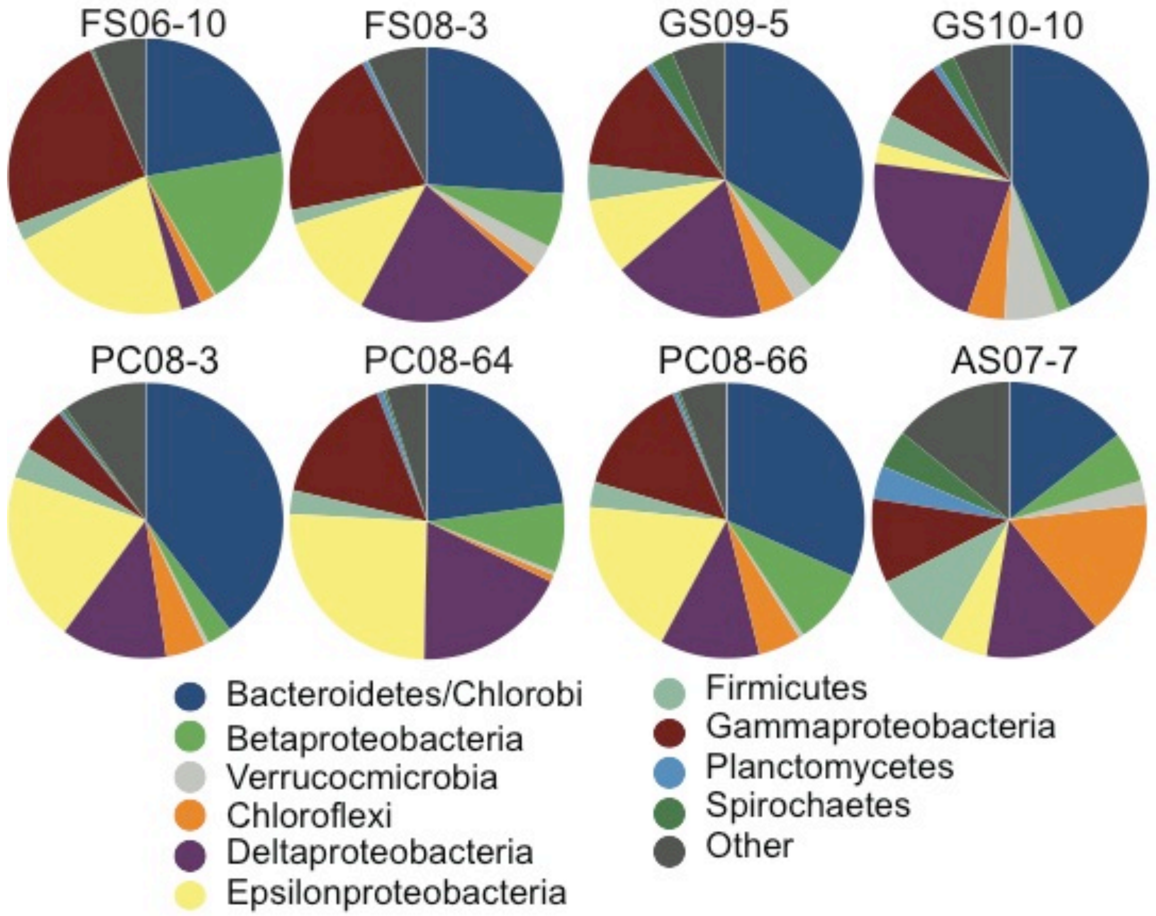

Figure S5. Taxonomic affiliation of all assembled metagenomic sequences affiliated with the Epsilonproteobacteria at the genus level (as defined in Campbell et al., 2006). Sequences affiliated with each genus were normalized to the total sequences in each metagenome. Other/Unclassified indicates all genus represented by less that 5% of the total. Sample designations are given in Table 1.

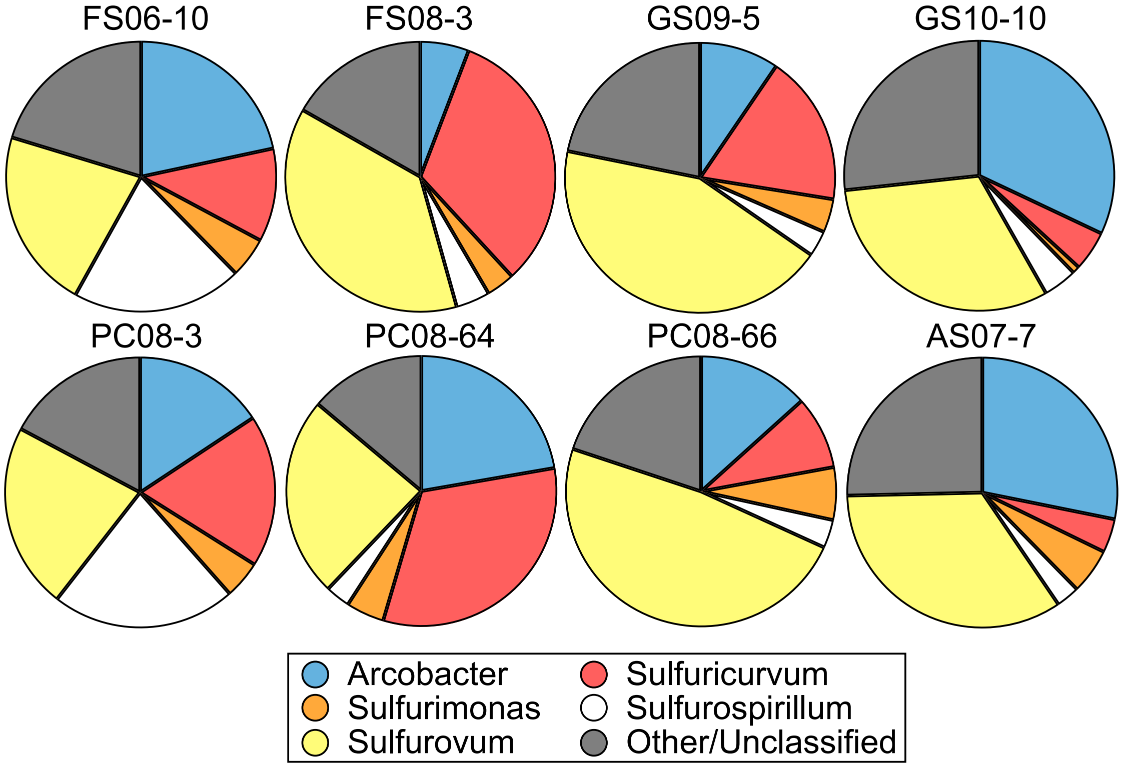

Figure S6. Taxonomic affiliation of Epsilonproteobacteria-affiliated ribosomal SP3 proteins classified at the genus level. A) Abundance of Epsilonproteobacteria-affiliated SP3 proteins based on the maximum likelihood phylogenetic tree in B). B) Epsilonproteobacteria-affiliated SP3 proteins mined from the metagenomes and from complete and draft genomes available from NCBI and IMG/M. Accession numbers or IMG designations are given in parentheses. Bootstrap support values >90 based on 1000 bootstrap samplings are shown for each node.

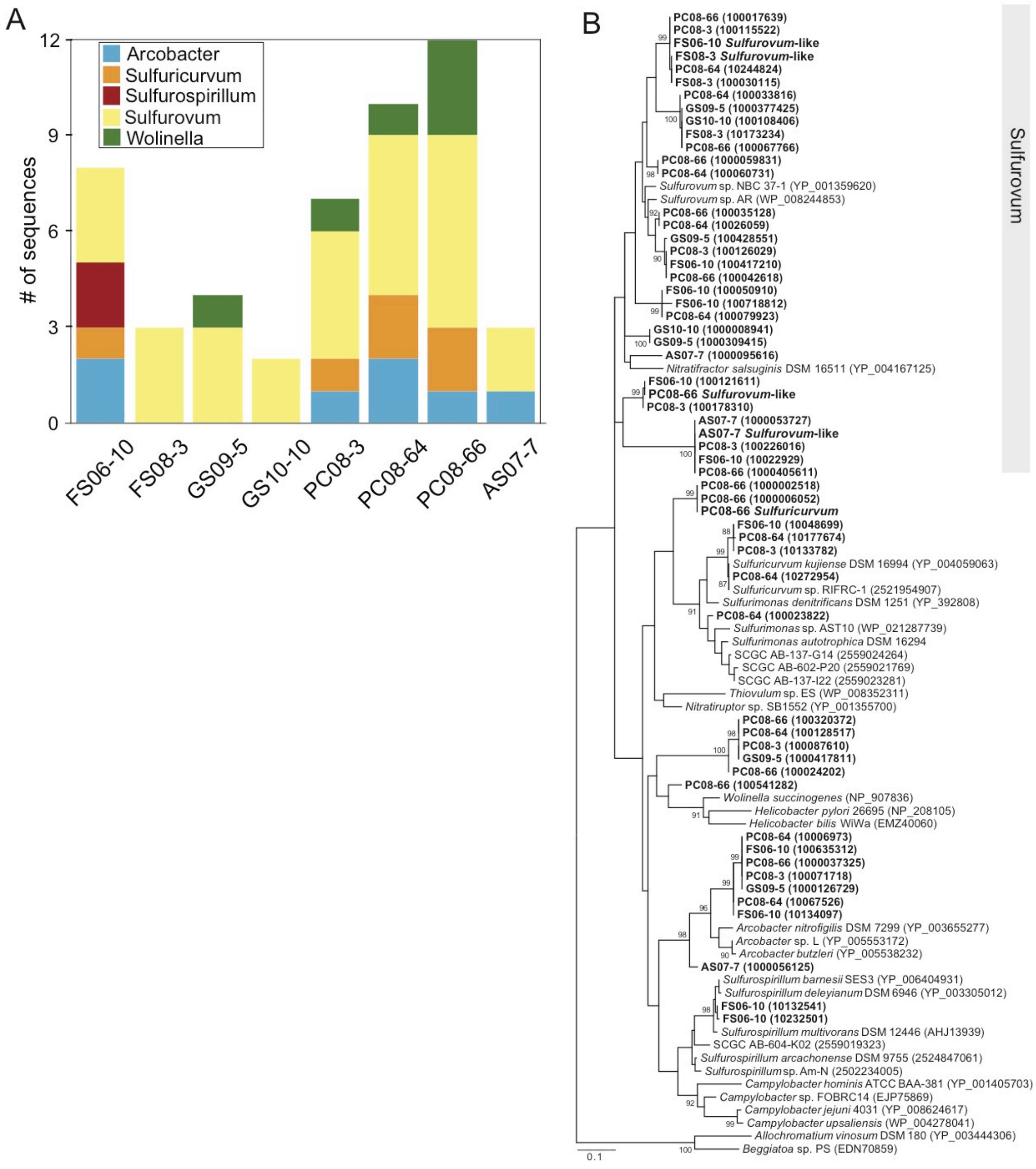

Figure S7. Phylogenetic diversity of full-length SoxB sequences mined from the eight assembled metagenomes. Maximum likelihood phylogenetic tree constructed from translated SoxB sequences identified in each dataset (as described in the Materials and Methods). Accession numbers or IMG designations are given in parentheses. Black cycles indicate bootstrap support values >90 based on 1000 bootstrap samplings.

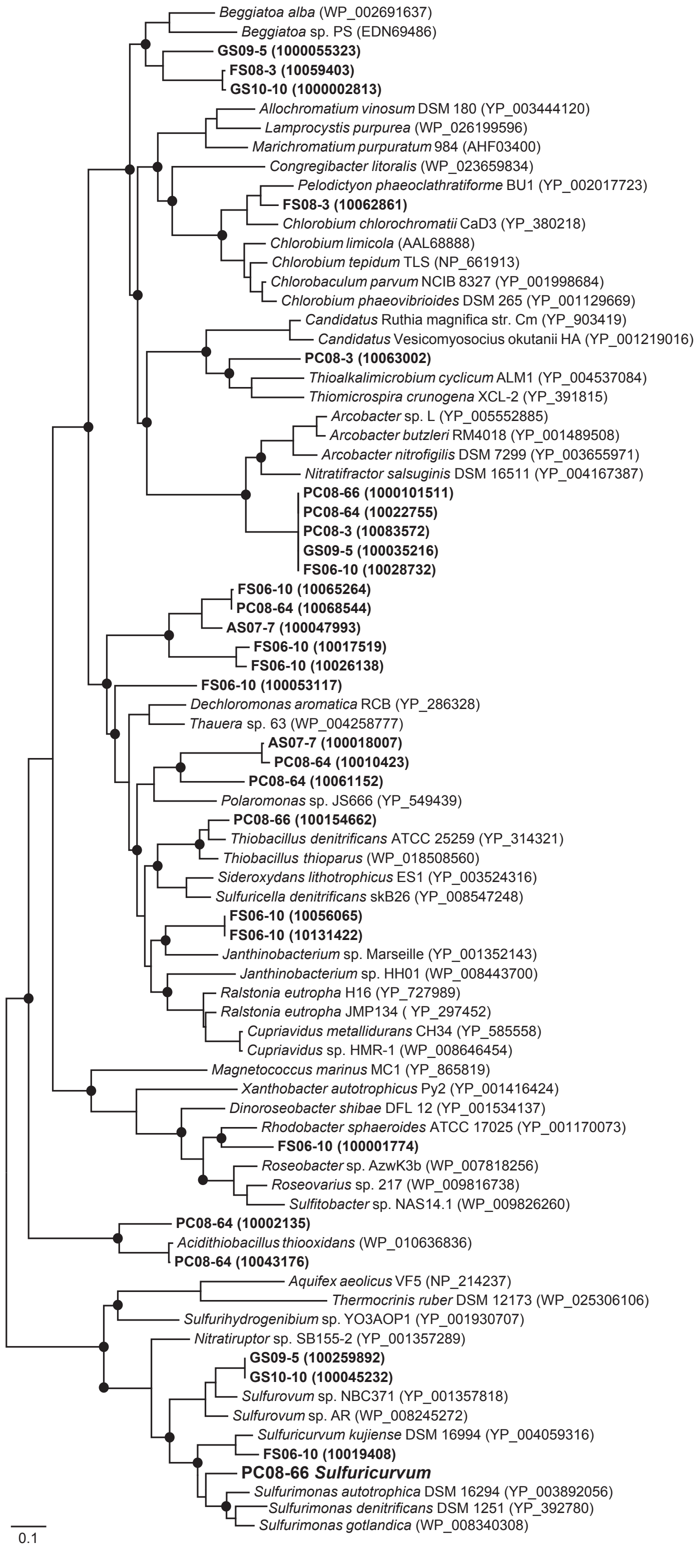

Figure S8. Phylogenetic diversity of Sqr in the metagenomes. Maximum likelihood phylogeny calculated from Sqr sequences mined from the metagenomes and the NCBI and IMG/M database. Classification of Sqr othologs is based on Gregersen et al., 2011. Numbers in parentheses indicate the number of Sqr sequences in the metagenomes assigned to each Sqr type.

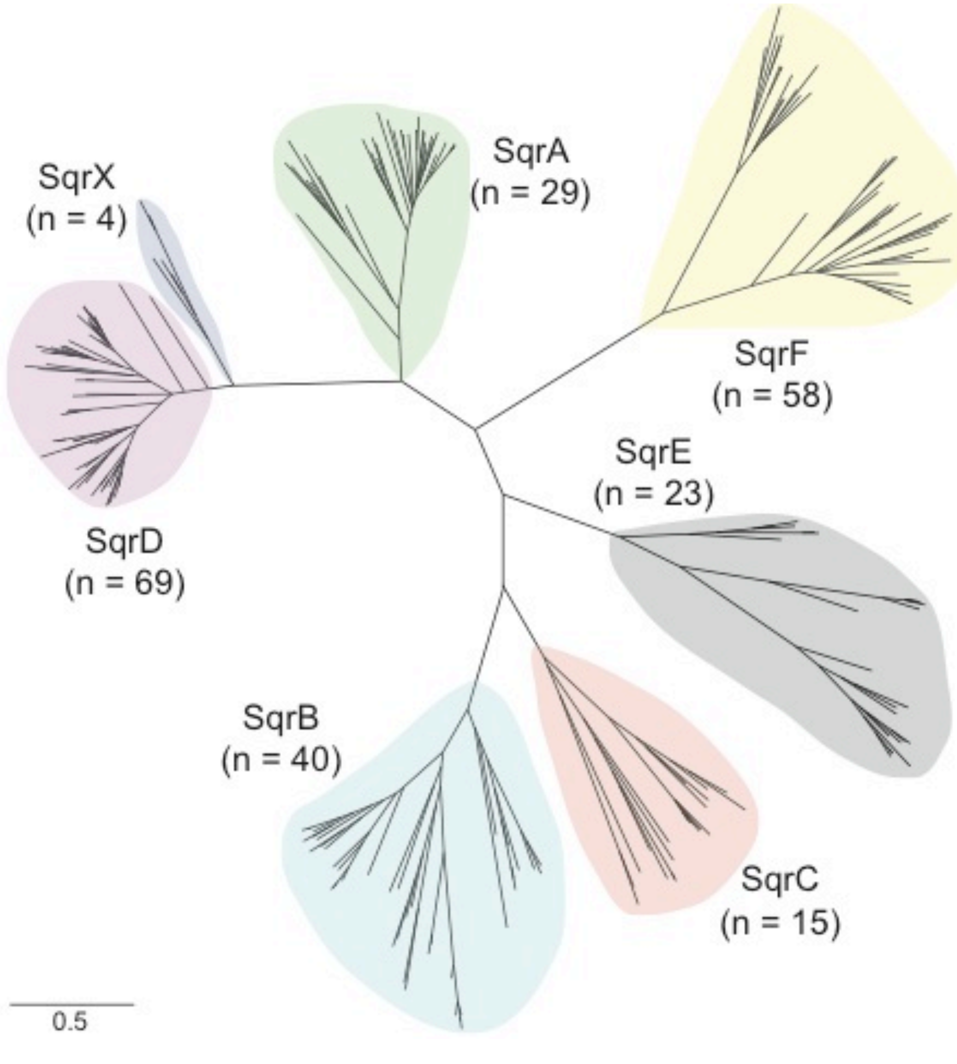

Figure S9. Phylogenetic diversity of rDsrAB in the metagenomes. Maximum likelihood phylogeny calculated from rDsrAB sequences mined from the metagenomes and the NCBI and IMG/M database (as described in the Materials and Methods). Accession numbers or IMG designations are given in parentheses (rDsrA, rDsrB). Bootstrap support values >90 based on 1000 samplings are indicated.

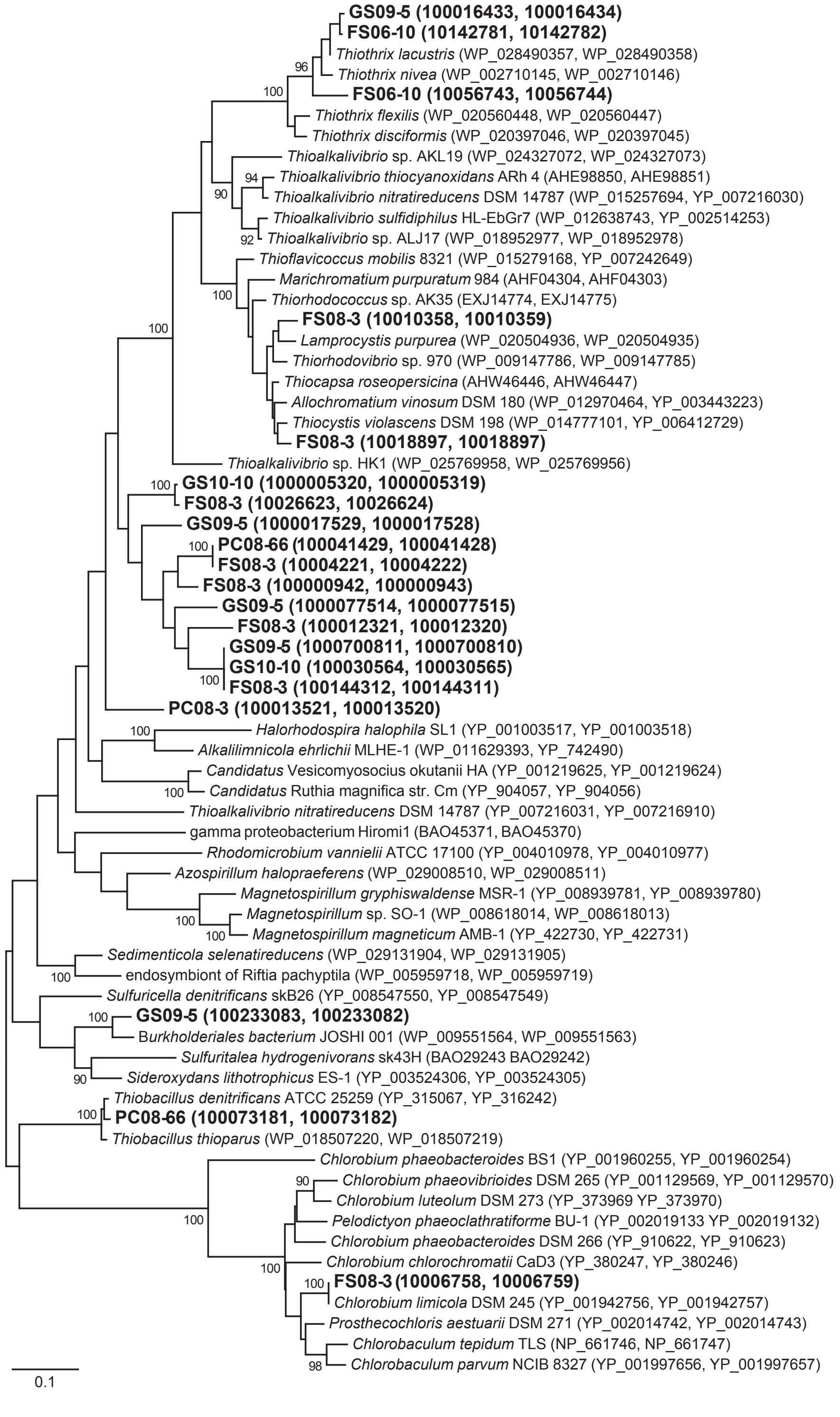

Figure S10. Phylogenetic diversity of AclA in the metagenomes. Maximum likelihood phylogeny calculated from AclA sequences mined from the metagenomes and the NCBI and IMG/M database (as described in the Materials and Methods). Accession numbers or IMG designations are given in parentheses. Black circles indicate bootstrap support values >90 based on 1000 samplings.

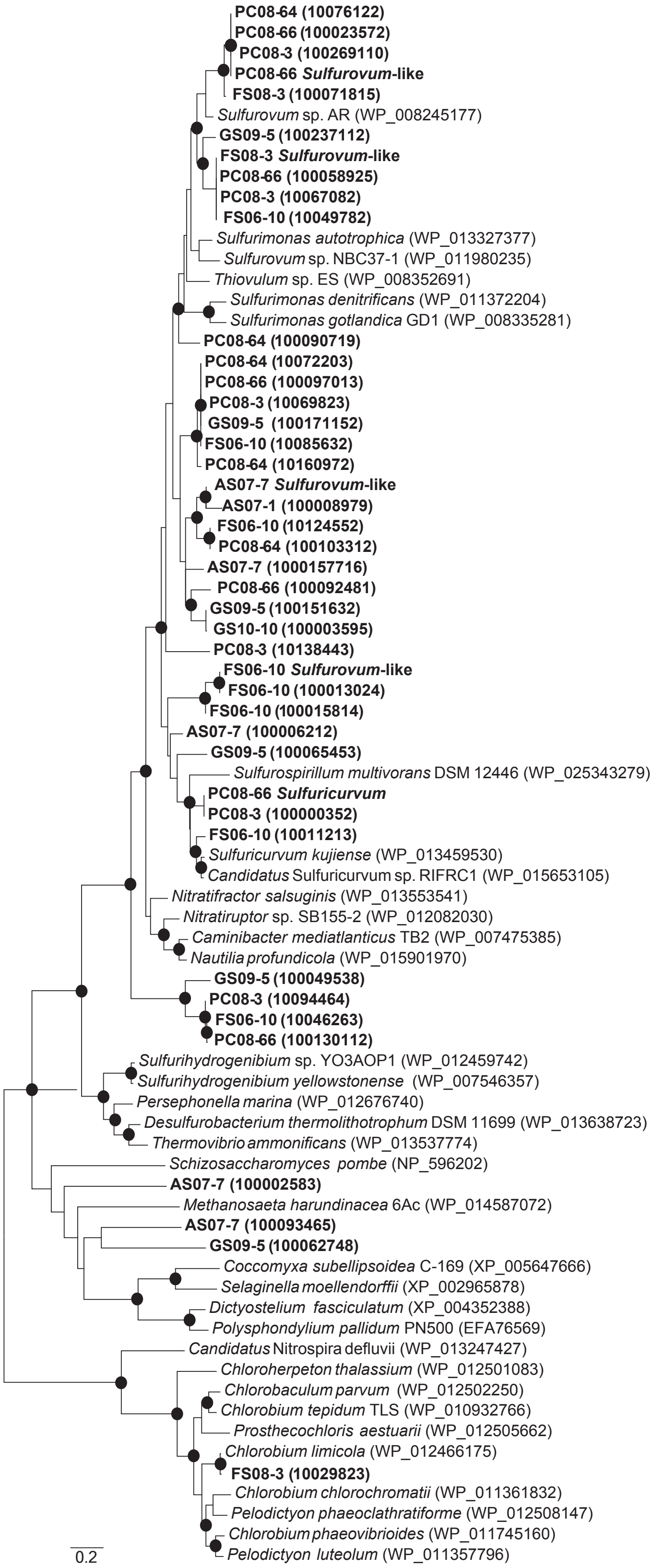

Figure S11. Maximum likelihood phylogenetic 16S rRNA gene tree of closely related Epsilonproteobacteria and the PC *Sulfuricurvum* genome. Accession numbers are given in parentheses. Bootstrap support values >90 based on 1000 samplings are noted.

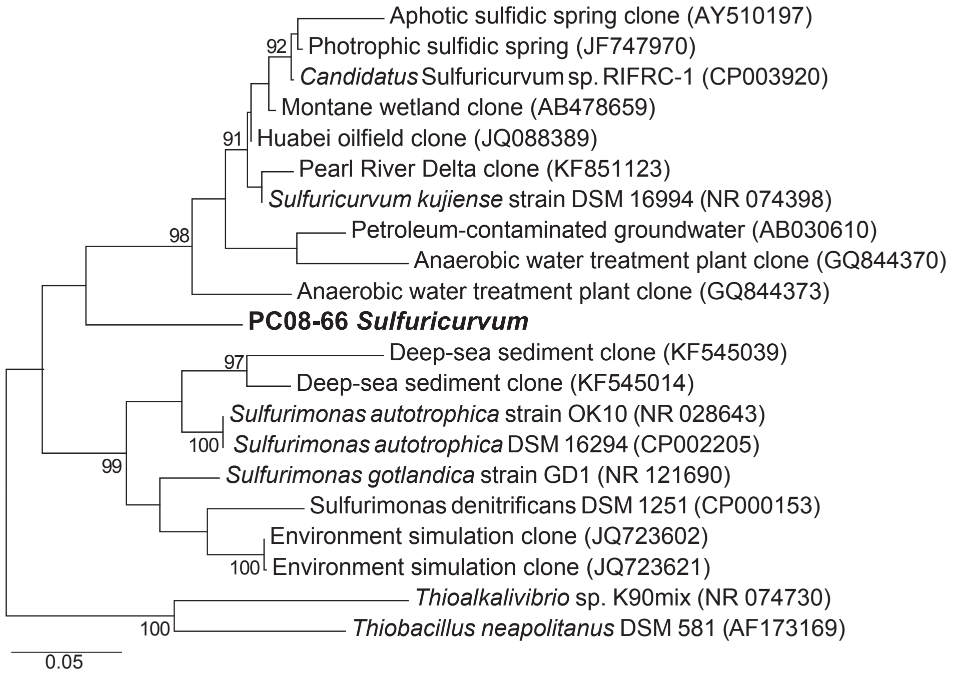

REFERENCES

Campbell, B.J., Engel, A.S., Porter, M.L., Takai, K. The e versatile ε-proteobacteria: key players in sulphidic habitats. Nature 4, 458–468. (doi: 10.1038/nrmicro1414)

Gregersen, L.H., Bryant, D.A., Frigaard, N-U. (2011). Mechanisms and evolution of oxidative sulfur metabolism in Green Sulfur Bacteria. *FMICB* **2**: 116. (10.3389/fmicb.2011.00116)
